# Systematic Identification of Molecular Signatures Dictating Therapeutic Effects of Clinically First‐Line Chemotherapy Regimens for Human Gastric Cancer Patients Based on Organoid Model

**DOI:** 10.1002/mco2.70656

**Published:** 2026-03-02

**Authors:** Jingwei Yang, Shuyue Qi, Yuan Gao, Jiansen Lu, Lin Deng, Xinglong Wu, Yifei Zhao, Yun Liu, Yanpeng Ma, Jiagui Song, Lixiang Xue, Lu Wen, Wei Fu, Fuchou Tang, Xin Zhou

**Affiliations:** ^1^ Biomedical Pioneering Innovative Center, Department of General Surgery Third Hospital Peking University Beijing China; ^2^ Peking‐Tsinghua Center for Life Sciences, Academy for Advanced Interdisciplinary Studies Peking University Beijing China; ^3^ Beijing Advanced Innovation Center for Genomics (ICG) Ministry of Education Key Laboratory of Cell Proliferation and Differentiation Beijing China; ^4^ Peking University Third Hospital Cancer Center Beijing China; ^5^ Center of Basic Medical Research, Institute of Medical Innovation and Research Peking University Third Hospital Beijing China; ^6^ Beijing Key Laboratory For Interdisciplinary Research in Gastrointestinal Oncology (BLGO) Beijing China; ^7^ School of Life Sciences Peking University Beijing China; ^8^ College of Animal Science and Technology Hebei Agricultural University Baoding Hebei China; ^9^ Changping Laboratory, Changping Laboratory Beijing China

**Keywords:** chemotherapy, drug screening, gastric cancer, molecular marker, patient‐derived organoid, tumor classification

## Abstract

Chemotherapy is the mainstay in the treatment of advanced gastric cancer (GC); yet, GC showed diverse responses to first‐line chemotherapy regimens and the underlying molecular basis is still not clear. Here, we established a system that combined organoid‐based chemotherapy regimen screening and transcriptome‐based evaluation to identify underlying molecular signatures of different responses to chemotherapy. We generated 19 GC patient‐derived organoids (PDOs) from surgically resected specimens with corresponding histological characteristics of parent tumors and tested all of the five most commonly used first‐line chemotherapy regimens. Based on the treatment responses, PDOs were classified into double‐sensitive, single‐sensitive, and not‐sensitive groups. PDOs that responded well to chemotherapy presented high expression levels of the P53 pathway genes and low expression levels of cell proliferative activity genes. Furthermore, the chemotherapy‐based tumor classification of GC was established. The GC tumor classification was verified by multi‐omics features from the TCGA dataset and public drug response datasets. In conclusion, this study systematically evaluated clinical chemotherapy regimens for GC and identified chemotherapy response‐associated molecular signatures based on human GC organoids, which are beneficial to the precise treatments of GC.

## Graphical abstract

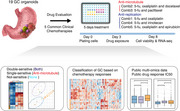

## 1 Introduction

Gastric cancer (GC) is one of the most heterogeneous diseases with high morbidity and mortality worldwide [1, 2]. Previous systematic researches have studied the malignancy and heterogeneity of GC at multi‐omics levels to explore intrinsic mechanisms and potentially effective treatments of GC. The Cancer Genome Atlas Program (TCGA) pointed out the detailed molecular classifications of GC with four main types: tumors positive for Epstein–Barr virus (EBV), microsatellite unstable tumors (MSI), genomically stable tumors (GS), and tumors with chromosomal instability (CIN) [3, 4]. The molecular classification was associated with pathological states well and provided important guidance for GC treatment. As for pathological subtypes, intestinal and diffuse subtypes from Lauren classification are the main types of GCs, and many bulk and single‐cell omics datasets revealed the similarities and differences between these types [1, 5].

Optimal therapeutic regimens for different subtypes of GCs are diverse. Although there are many novel therapies for GC emerging, chemotherapy remains the primary treatment strategy for GC patients as a supplement or an alternative to surgery currently [1, 5, 6]. There still are unclear and important mechanisms for responses of several chemotherapies, especially for the combination therapies of multiple small molecule drugs, which are the main parts for clinical chemotherapies for GC. Due to the diversity of the GC subtypes and the limited understanding of clinical treatment effects, chemotherapy regimens are not always effective or optimal, and how to choose different chemotherapy regimens is mainly based on the clinician's experiences [7]. Hence, it is quite important for GC treatments to explore predictive biomarkers and molecular classification systems to guide the accurate selection of chemotherapy regimens [8], emphasizing the relationship between subtypes of GCs and responses to different chemotherapy regimens, especially the underlying molecular signatures.

In recent years, patient‐derived organoids (PDOs) have been developed and applied in many aspects of cancer research [9, 10, 11]. PDOs can faithfully represent molecular and pathological signatures of the tumors, which even mimic the process of tumor initiation and evolution. PDOs can be cultured for a long time, and organoid cells can robustly maintain tumor features [12, 13]. Based on these characteristics, drug screening and testing trials have been performed for different types of tumors on PDOs, including GCs [14, 15, 16]. Several previous studies have established biobanks for gastric cancer PDOs and performed corresponding drug screening tests [17, 18, 19, 20], confirming the reliability of PDOs in GC research. However, few PDO studies focused on the molecular signatures associated with responses to first‐line chemotherapy regimens for GC.

**FIGURE 1:**
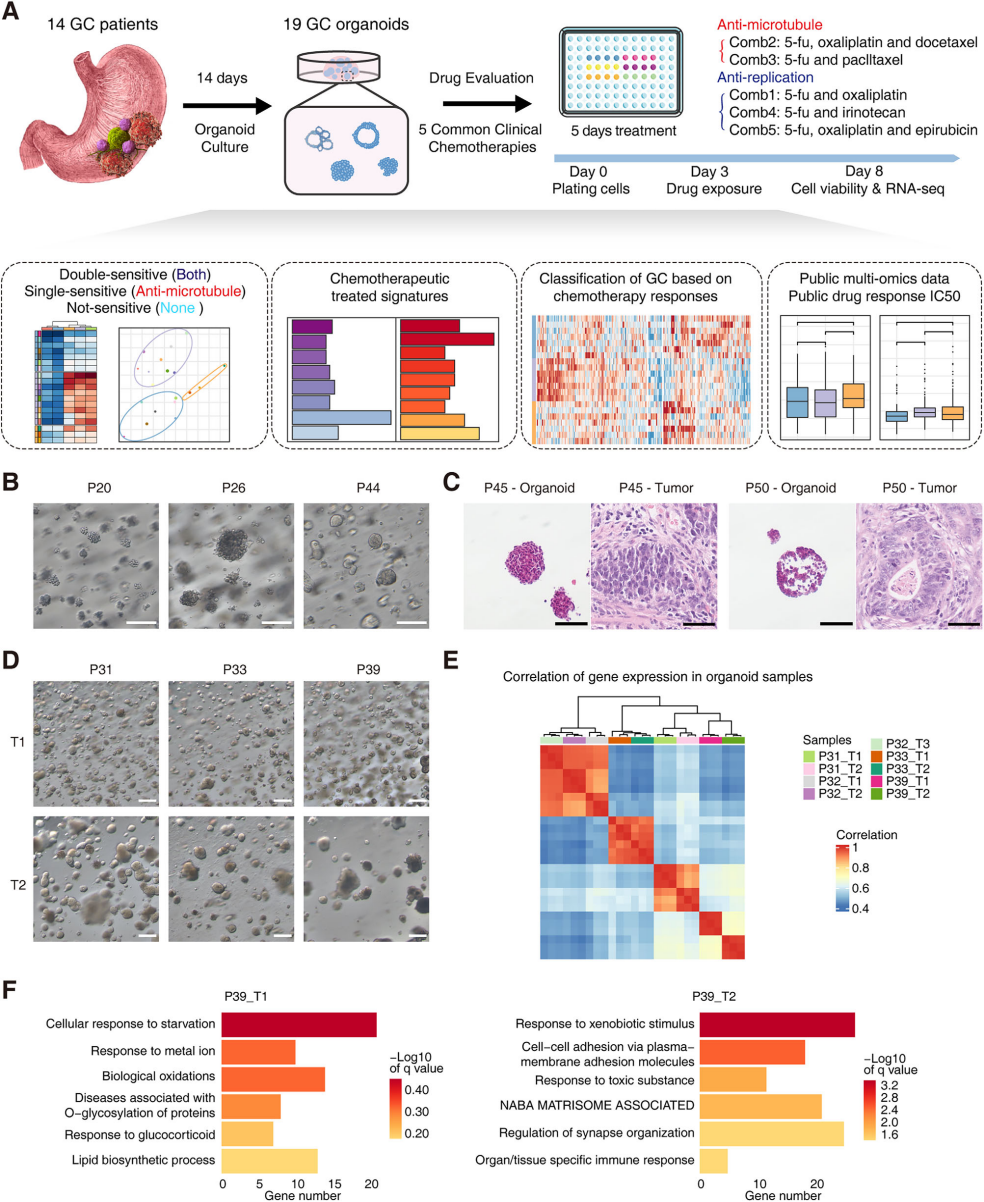
GC PDO establishment and diverse signature characterization. (A) The workflow of GC PDO establishment, drug treatment, classification of chemotherapy response groups, altered transcriptome analyses, and public data verification. (B) The bright field images of PDOs from P20, P26, and P44. Scale bar: 200 µm. (C) H&E staining of the primary tumors and organoids from P45 and P50. Scale bar: 100 µm for PDOs and 50 µm for primary tumors. (D) The bright field images of PDOs from two tumor sites of P31, P33, and P39. Scale bar: 300 µm. (E) Correlation of the multi‐site PDOs in the general transcriptome level. (F) The differentially enriched signatures of P39_T1 and P39_T2. The terms were enriched using Metascape.

To explore the connections between GC patient subtypes and responses to different drug treatments, we systematically evaluated the responses of the five most commonly used first‐line chemotherapy regimens based on 19 PDO lines established from GC patients [21, 22]. The gene expression signatures of chemotherapy treatments were systematically analyzed and used as the main assessment criteria. In brief, this study based on tumor organoids described the detailed gene expression signatures, which are presented by multiple GC samples after currently clinically most commonly used chemotherapy treatments, and it also provided the classifications and molecular signatures of different responses to the treatments (Figure 1A). Particularly, the identification of three prognosis‐related response groups was extended to larger cohorts including CCLE and TCGA, which indicates their universality in GC.

## 2 Results

### 2.1 Establishment of GC Organoids and Treatment Evaluation of Five Clinically Most Commonly Used Chemotherapy Regimens

To evaluate different clinical chemotherapies of GCs, we established GC PDOs based on the previously published protocols with several modifications of the culture medium and methods (see Section 4). The rough success rate is about 70% for collected GC samples in our study, and the average culturing period is about 14 days (Figure 1A). In total, 19 primary GC tumor samples with multiple sites from 14 surgically resected GC specimens were collected, and the corresponding GC organoids were derived maintaining the long‐term expansion capacities (Table S1). Among these GC samples, seven are from gastric antrum and the other seven are from gastric corpus. Of these 14 GC patients, five are females and nine are males. Six patients’ tumors are poorly differentiated, whereas eight are differentiated. Six of them are diffuse GC and eight are intestinal GC. Two of them are MSI type and others are microsatellite stability (MSS) type or undefined. These patients included essentially all major subtypes of GC with multiple pathological signatures, indicating the representative feature of this cohort. The morphologies of organoids from different patient tumor tissues were diverse, which presented the inter‐patient and intra‐patient heterogeneities of GC organoids (Figure 1B and Figure S1A). The H&E staining images of organoids in vitro and corresponding patient tumor tissues in vivo pointed out that the histological signatures were in general maintained between the in vitro cultured organoids and original tumors in vivo (Figure 1C and Figure S1B).

Here, nine multi‐site tumor regions from four patients were captured in order to reveal the intra‐tumoral heterogeneities of GCs. We noticed that tumor sites from P31, P32, and P33 showed quite consistent morphology, while two sites from P39 showed different morphologies through the brightfield images, which indicated the intra‐tumoral heterogeneities in local tumor tissues (Figure 1D and Figure S1C). As for the corresponding transcriptome states, tumor sites from P32 and P33 showed similar gene expression patterns, while two tumor sites from P39 presented distinct gene expression patterns (Figure 1E). Furthermore, we identified the differentially expressed signatures between P39_T1 and P39_T2, with significantly higher expression of the differentially expressed genes (DEGs) such as *SLO2A1*, *LGR5*, *CFTR*, *MYH4* in P39_T1 and *NQO1*, *TFF1*, *S100P*, *ALDH1A1* in P39_T2 (Figure S1D). P39_T1 presented signatures of normal digestive functions such as cellular response to starvation, response to metal ion, and response to glucocorticoid, while P39_T2 presented signatures of responses to external stimulus functions including response to xenobiotic stimulus, cell–cell adhesion via plasma‐membrane adhesion molecules, and response to toxic substance (Figure 1F). These differential molecular signatures revealed the advantages of the organoid system in faithfully maintaining intra‐tumoral heterogeneities.

Using these PDOs, we evaluated the cancer cell‐killing effects of five clinically first‐line chemotherapy regimens for GCs. All these five clinical chemotherapy regimens contained 5‐fluorouracil (5‐fu), and the detailed drug combinations are as follows: Combination1 (Comb1), 5‐fu and oxaliplatin; Combination2 (Comb2), 5‐fu, oxaliplatin and docetaxel; Combination3 (Comb3), 5‐fu and paclltaxel; Combination4 (Comb4), 5‐fu and irinotecan; Combination5 (Comb5), 5‐fu, oxaliplatin and epirubicin. We performed treatments with 5 days of drug exposure and used cell viability as the indicator of cancer cell–killing effects (Figure 1A). For all these five treatments, significantly decreased cell viabilities compared with untreated control were shown, which presented the effective tumor killing effects for all the treatments (Figure S1E). We also tested different drug treatment durations ranging from 1 to 5 days for organoids of P16 and P24. The high consistencies of different drug treatment durations across all the treatments were revealed by DEGs and differentially expressed features, and the changes of response‐related molecular signatures were consistent between different treatment durations for the same chemotherapy regimen (Figure S2A,C,D). For example, in P16, cell cycle and metabolism‐related pathways such as G2M checkpoint, chromosome organization, and small molecular catabolic process were suppressed gradually along different treatment days, whereas interferon‐ and cell death‐associated signatures were gradually increased (Figure S2A). The principal component analysis (PCA) showed that the variation captured by PC1 reflected a continuous shift in molecular signatures from day 1 to day 5 of treatment (Figure S2B). Similar changes were also presented in P24 (Figure S2D). These findings confirm the robustness of our drug‐treatment system and indicate that a 5‐day treatment window is appropriate for endpoint analyses. The progressive transcriptomic changes following drug treatment further support the consistency of our treatment strategy and the validity of our GC PDO–based evaluation approach.

### 2.2 Identification of Double‐, Single‐, and Not‐Sensitive Sample Groups According to the Heterogeneous Responses to Anti‐Microtubule and Anti‐Replication Treatments

**FIGURE 2:**
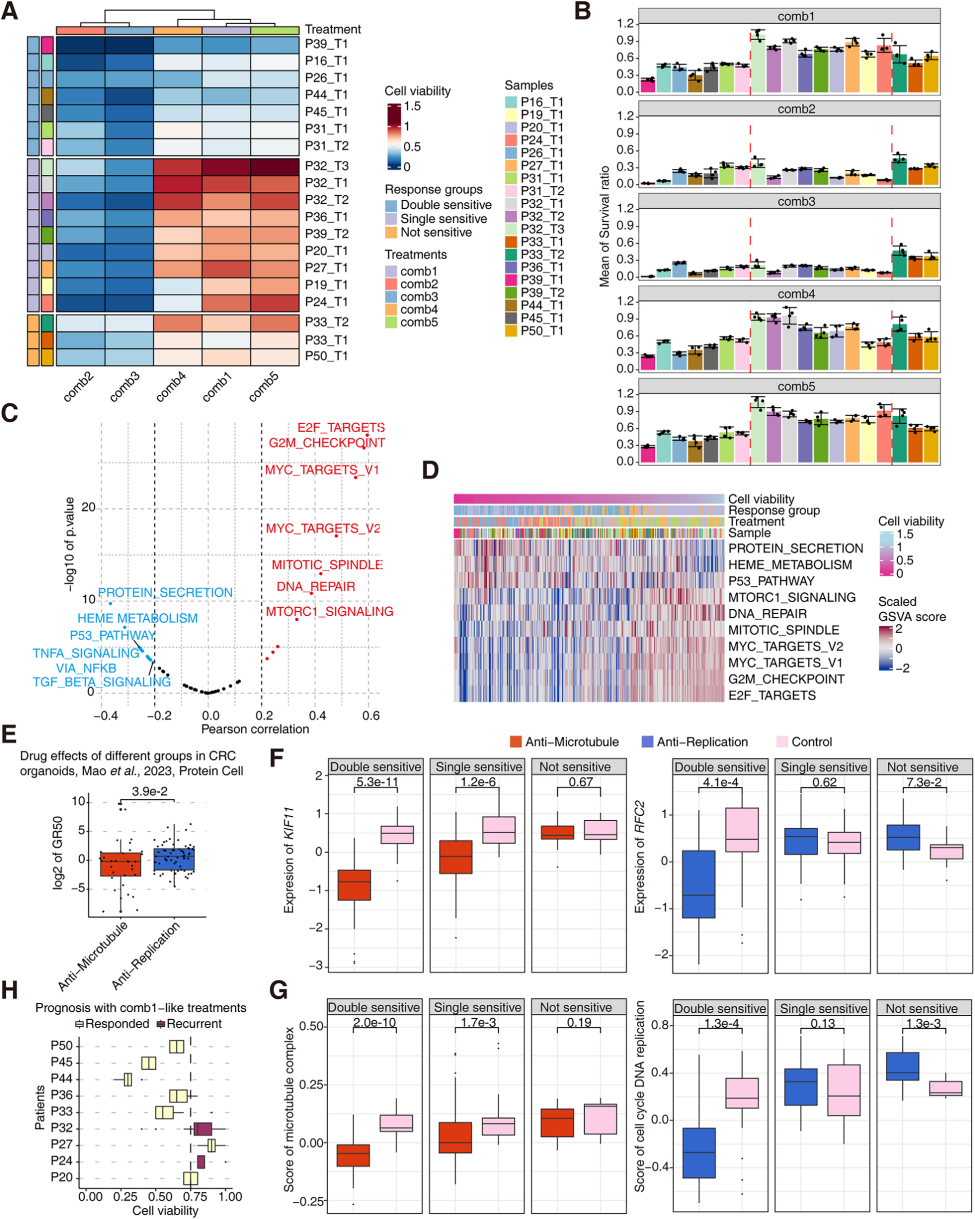
The classification of GC PDOs based on treatment effects. (A) The classification of 19 PDOs based on cell viability after different types of treatments. (B) The mean survival ratios of all PDOs (*n* = 4) after five chemotherapy combination treatments. (C) Correlation of hallmark geneset scores and cell viability after treatments. (D). Expression of hallmark genesets of all the treated PDOs arranged by the value of cell viability. Parts of legends in the figure refer to (A). (E) Drug effects of the anti‐microtubule and anti‐replication treatments in CRC organoids from another published dataset, revealed by GR50 values (*n* = 32 in the anti‐microtubule group and *n* = 64 in the anti‐replication group). (F) Expression of *KLF11* and *RFC2* in three response groups after the anti‐microtubule and anti‐replication treatments (*n* = 42 in the double‐sensitive group, *n* = 54 in the single‐sensitive group, and *n* = 18 in the not‐sensitive group from the anti‐microtubule treatment; *n* = 63 in the double‐sensitive group, *n* = 81 in the single‐sensitive group, and *n* = 27 in the not‐sensitive group from the anti‐ replication treatment; *n* = 21 in the double‐sensitive group, *n* = 27 in the single‐sensitive group, and *n* = 9 in the not‐sensitive group from the control group). (G) Expression scores of the microtubule complex term and the cell cycle DNA replication term in three response groups after the anti‐microtubule and anti‐replication treatments (*n* = 42 in the double‐sensitive group, *n* = 54 in the single‐sensitive group, and *n* = 18 in the not‐sensitive group from the anti‐microtubule treatment; *n* = 63 in the double‐sensitive group, *n* = 81 in the single‐sensitive group, and *n* = 27 in the not‐sensitive group from the anti‐replication treatment; *n* = 21 in the double‐sensitive group, *n* = 27 in the single‐sensitive group, and *n* = 9 in the not‐sensitive group from the control group). (H) The cell viability of PDOs treated with Comb1 and the corresponding relationship with the patient's prognosis (*n* = 4).

Based on these 19 organoids and drug treatment strategies, we used cell viability and transcriptome signatures after drug treatment to elucidate tumor‐killing effects and detailed mechanisms of these five clinical chemotherapy regimens. Using average values of cell viability per treatment from each sample (three replicates per treatment for each sample, in total five treatments and 19 PDOs), hierarchical clustering split treatments into two groups and clustered these samples into three groups (Figure 2A,B). As for treatment groups, Comb2 and Comb3 included docetaxel or paclitaxel, which both bind to tubulin, stabilize microtubules, and cause cell cycle stasis in G2/M phase [23, 24]. However, Comb1, Comb4, and Comb5 only contained drugs whose mechanism of action (MOA) is mainly to inhibit DNA replication and transcription. We termed these two groups as the anti‐microtubule group (Comb2 and Comb3) and the anti‐replication group (Comb1, Comb4, and Comb5), respectively (Table S2). Combined with the classification of treatments, the organoid response patterns were identified as the double‐sensitive group (sensitive to both anti‐microtubule treatments and anti‐replication treatments), the single‐sensitive group (only sensitive to anti‐microtubule treatments while not responding to anti‐replication treatments), and the not‐sensitive group (insensitive to both anti‐microtubule treatments and anti‐replication treatments). According to the average cell viability values of the anti‐microtubule and anti‐replication treatments, PDOs were also aggregated clearly (Figure S3B). It should be noted that a couple of multi‐site tumor PDOs from the same patient were located in the same group, and only P39_T1 and P39_T2 were in the double‐sensitive group and the single‐sensitive group, respectively, which also corresponds to the different molecular signatures of these two organoid samples (Figure 1F). As for individual organoid samples, corresponding drug responses to different chemotherapy regimens were consistent with the above classifications, respectively (Figure S3C).

We also identified the altered signaling pathways associated with cell viability after drug exposure. The hallmark signatures such as E2F targets, G2M checkpoint, and mitotic spindle were positively correlated with cell viability, whereas the hallmark signatures such as protein secretion, HEME metabolism, and p53 pathway were negatively correlated with cell viability (Figure 2C). Along with the cell viability that gradually increased in the samples, the scores of these signaling pathways and corresponding genes gradually increased or decreased accordingly, and these identified signatures were in accordance with activators or suppressors of tumor progression as expected [25] (Figure 2D and Figure S3A). Of them, p53 signaling pathway is one of the most important tumor‐suppressing pathways involved in essential cellular processes such as cell division, maintenance of genomic stability, apoptosis, autophagy, and immune response [26]. Heme metabolism was reported to be associated with drug resistance in previous studies [27, 28, 29]. The differentially regulated signatures associated with cell viability revealed the complexity of drug responses of GCs.

According to cell viability values from merged samples per treatment, it seemed that the overall effects of anti‐microtubule treatments were better than anti‐replication treatments based on our GC organoid system (Figure 2B, Figure S4A,B). Colorectal cancer (CRC) and GC share the common chemotherapy regimens in clinical practice [30, 31, 32]. We used another dataset from our previous study involving drug screening based on human CRC organoids, and some of the tested drugs targeting microtubules or DNA replication [33]. In the primary round of drug screening in vitro, excluding those powerful tumor‐killing drugs, anti‐microtubule drugs had lower cell viability values than anti‐replication drugs in both of the two CRC organoids (Figure S4C). In the secondary round of drug screening over a long period, we also found that the GR50 values (a modified measurement of IC50 with reduced cell growth influences) of anti‐microtubule drugs were significantly lower than anti‐replication drugs [34] (Figure 2E). Among these four organoid samples of the CRC dataset with drug GR50 values from the secondary round of drug screening, P2 and P3 organoids presented the slightly lower average GR50 of anti‐microtubule drugs, while P4 and P5 did not show differences between anti‐microtubule drugs and anti‐replication drugs (Figure S4D). The clinical information showed that tumor sites of P4 and P5 had clear lympho‐vascular invasion, which indicated that the potential metastatic tendency may influence treatment effects of chemotherapies.

To further verify the underlying mechanisms, we screened gene expression signature scores in different response groups compared with the untreated controls. Key functional genes associated with microtubule formation including *KIF11*, *BIRC5* and *HAUS1* showed lower expression than untreated controls in the double‐sensitive group and the single‐sensitive group, with no differences in the not‐sensitive group (Figure 2F and Figure S4E). DNA replication–related genes such as *RFC2*, *POLD1*, and *POLD3* only had the low expression levels in the double‐sensitive group, with no changes in the single‐sensitive group and the not‐sensitive group (Figure 2F and Figure S4E). In addition to vital genes involved in these processes, signaling pathways such as microtubule complex, microtubule binding, and tubulin binding were downregulated in both the double‐sensitive group and the single‐sensitive group, while cell cycle DNA replication, DNA replication, and DNA synthesis in DNA repair functions were downregulated only in the double‐sensitive group (Figure 2G and Figure S4G,H). These identifications on MOAs verified the actual influences of these two types of treatments on organoids in these three response groups.

We also integrated the clinical treatments and responses of individual patients with our data in organoids, and identified the consistency of treatment responses and clinical outputs (Figure 2H). Nine of the patients received the treatment Combination 1. Our results in the PDO model could predict the clinical outcomes in eight patients. Tumor recurrence occurred in two of them, and both of these two patients had high cell viabilities (more than 0.75, which means the low drug killing effect) compared with other patients, while only one of the patients with the good response had the high cell viability and other patients with good response all presented low cell viabilities in our treating trials based on organoids.

### 2.3 Treatment Response‐Related Effects Revealed by Prominent Pathways in All the Treated PDOs

**FIGURE 3:**
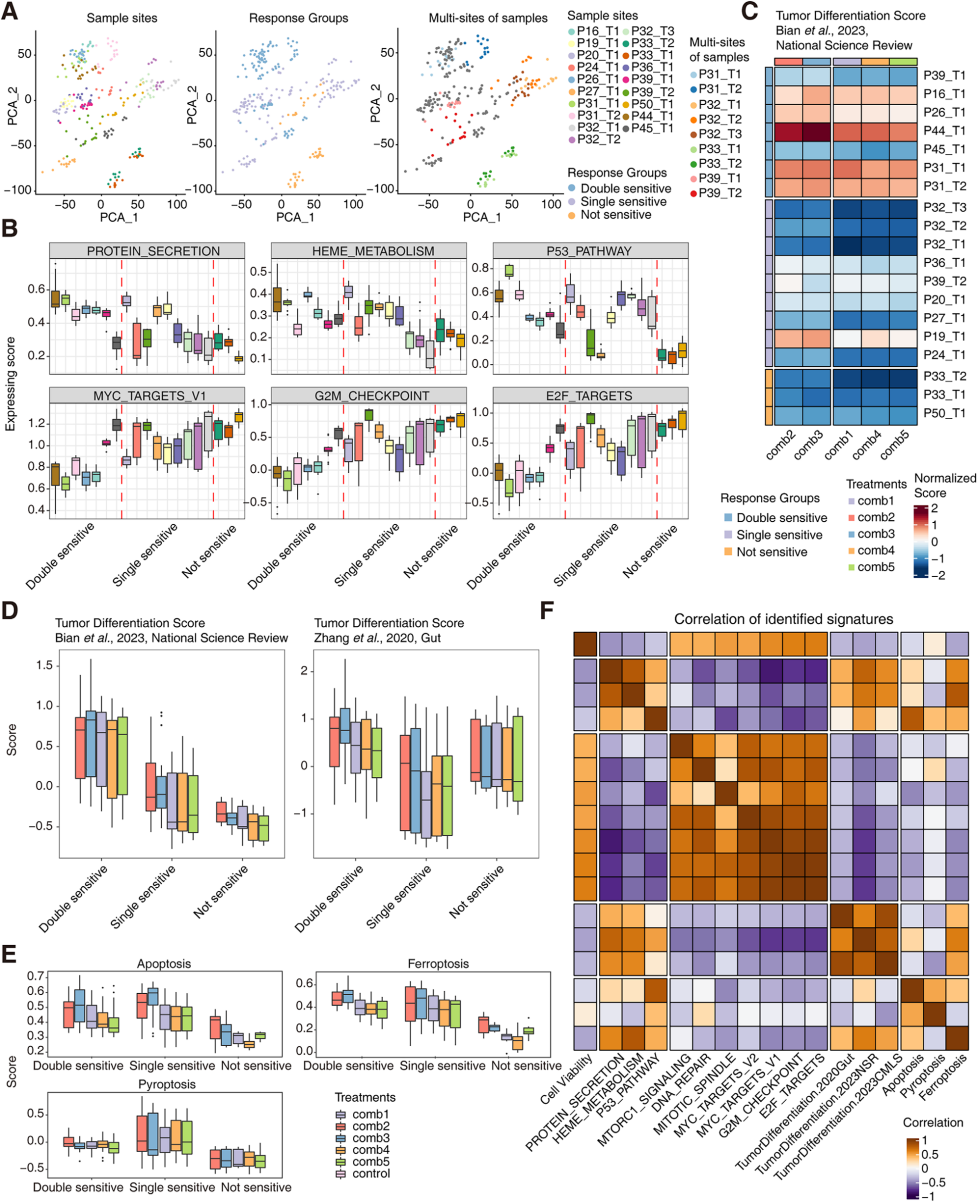
Transcriptome signatures associated with cell viability after treatment. (A) The scatterplot of PCA about all the treated PDOs with different annotations, including sample sites, response groups, and multi‐sites. (B) The expression scores of geneset signatures in different PDOs from different response groups (*n* = 3). (C) The heatmap shows the expression scores of the tumor differentiation geneset from a published dataset in different PDOs from different response groups. (D) The boxplots show the expression scores of the tumor differentiation genesets from another two published datasets in different PDOs from different response groups (*n* = 105 in the double‐sensitive group, *n* = 135 in the single‐sensitive group, and *n* = 45 in the not‐sensitive group). (E) The boxplots show the expression scores of apoptosis, ferroptosis, and pyroptosis genesets across different samples from different response groups (*n* = 105 in the double‐sensitive group, *n* = 135 in the single‐sensitive group, and *n* = 45 in the not‐sensitive group). (F) The correlation relationship of expression scores of different enriched terms and cell viabilities after treatments.

Although the overall inhibiting effects of the treatments were clear, the identification of response groups indicated that different signatures associated with subgroups of these treated organoids should be clarified. Here, we performed the dimensionality reduction analysis with PCA for all the chemotherapy‐treated organoids. In general, organoids were clustered by patient sources rather than by treatments, and multi‐site tumor organoids were clustered together (Figure 3A and Figure S5A). The PC2 dimension of the PCA analysis roughly showed the molecular effects of different response groups, with the arrangement of the not‐sensitive group, the single‐sensitive group, and the double‐sensitive group along the PC2 dimension (Figure 3A). Clinical information including GC tumor types (diffuse or intestinal types), GC tumor locations (gastric antrum or gastric corpus) and several genetic factors (*TP53* mutations, EBV‐positive, and HER2 expression) seem not to be associated with the response groups (Figure S5A).

Furthermore, cancer‐associated signatures and several previously identified signatures were analyzed to evaluate the underlying mechanisms. Protein secretion, heme metabolism, and P53 pathway were enriched in the double‐sensitive group, while MYC targets, G2M checkpoint, E2F targets, mitotic spindle, and DNA repair were enriched in the not‐sensitive group (Figure 3B and Figure S5B,C). These different signatures were in general consistent with the results of the correlation of hallmark genesets and cell viability after chemotherapy treatments. Then, we used several GC tumor differentiation genesets from three published GC single‐cell datasets [35, 36, 37]. The analysis combining all these three tumor differentiation genesets showed the consistent results, which is that tumor differentiating state signature was significantly enriched in the double‐sensitive group (Figure 3C and Figure S5D). Even in the same response group, samples treated with Comb2 and Comb3 presented a higher score than Comb1, Comb4, and Comb5 (Figure 3D and Figure S5E). Finally, we scored different death type genesets including apoptosis, ferroptosis, and pyroptosis (Table S3). The apoptosis and ferroptosis signatures were slightly higher in anti‐microtubule treatments than anti‐replication treatments, and presented higher scores in the double‐sensitive group and the single‐sensitive group than the not‐sensitive group (Figure 3E and Figure S5F). These results indicated that the main cell death types were apoptosis and ferroptosis after these chemotherapy treatments. In summary, clear signatures were associated with treatment responses, which indicated the intrinsic mechanisms of these treatments (Figure 3F).

### 2.4 Treatment Response‐Related Effects Revealed by Molecular Changes of Treatments and Response Groups Compared With Control

**FIGURE 4:**
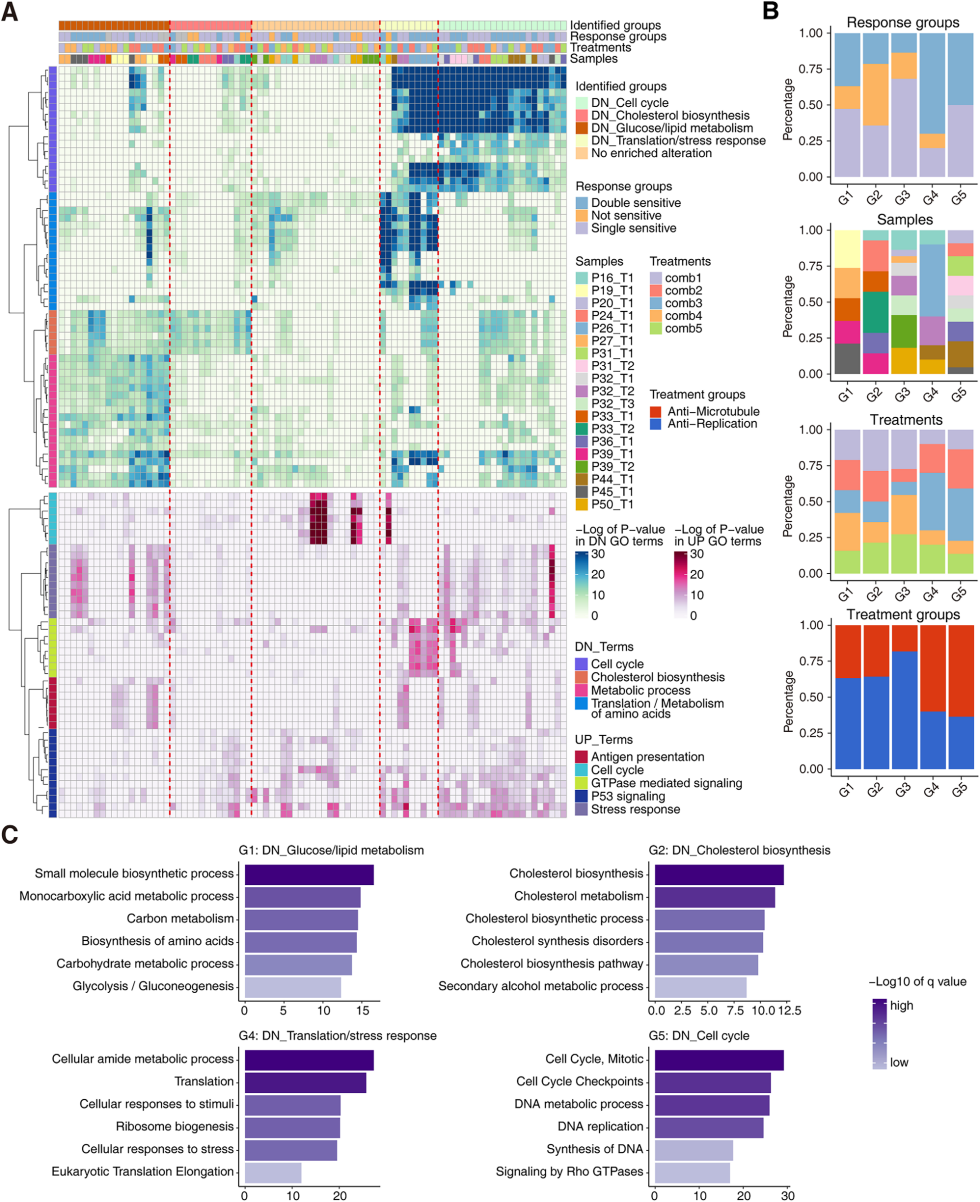
Classification based on downregulated and upregulated enriched GO terms. (A) The enriched differentially expressed terms of all the treated PDOs. The top bars present the corresponding sample annotations and the unsupervised hierarchical clustering of all the treated PDOs. The upper heatmap presents the downregulated terms, and the bottom heatmap presents the upregulated terms. (B) The proportions of different classified groups in diverse annotating groups, including response groups, samples, treatments, and treatment groups. (C) The enriched gene terms of different classified groups from the unsupervised hierarchical clustering.

In addition to differential molecular signatures and response effects of treated organoid samples, the molecular perturbations and influences of treated samples compared to untreated controls disclosed the embedded mechanisms of these five treatments (Table S4). Using the negatively and positively enriched gene functions of each treatment compared with untreated controls, we performed hierarchical clustering and clustered organoid samples into five patterns (G1–G5) with differentially changed signatures (Figure 4A,C). G1 presented the downregulation of glucose and lipid metabolism, with detailed enriched functions such as small molecule biosynthetic process and glycolysis/gluconeogenesis. G2 presented the downregulation of cholesterol biosynthesis. G3 presented minor alterations based on DEGs and essentially no enriched functional signatures. G4 presented the downregulation of translation process and stress responses. G5 presented the inhibition of cell cycle functions. As for these five patterns, anti‐microtubule treatments were enriched in G4 and G5, while the not‐sensitive group was enriched in G1, G2, and G3 (Figure 4B). Detailed changes of individual organoid samples with different drug combination treatments were demonstrated, respectively, which were in accordance with the above five patterns (Figure S6A–D).

**FIGURE 5:**
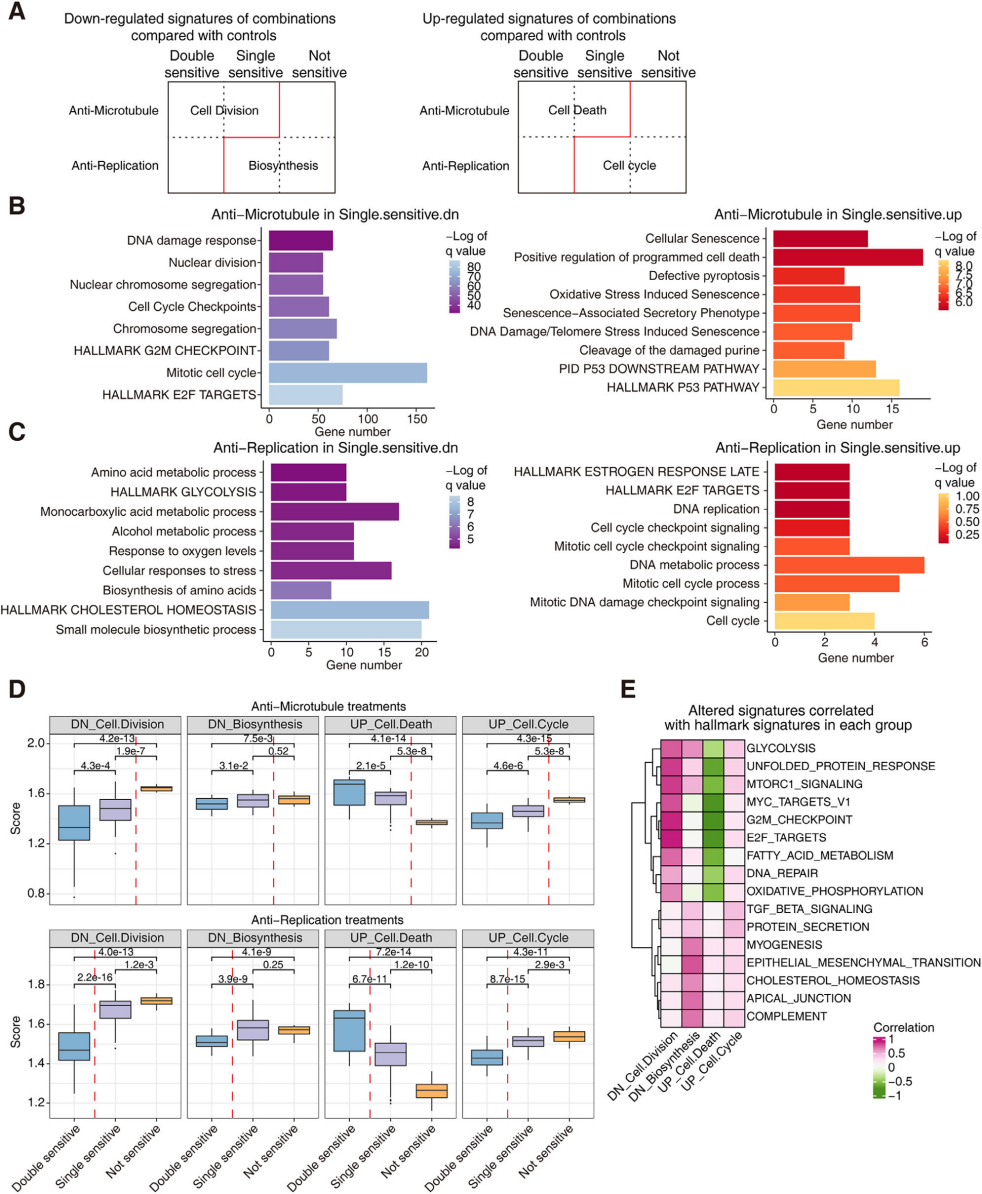
Differentially expressed signature genes of the anti‐microtubule and anti‐replication treatments in the three response groups. (A) The schematic diagram shows the downregulated and upregulated enriched terms of the anti‐microtubule and anti‐replication treatments across the double‐sensitive group, the single‐sensitive group, and the not‐sensitive group. (B) The enriched downregulated and upregulated gene terms of the anti‐microtubule treatments in the single‐sensitive group. (C) The enriched downregulated and upregulated gene terms of the anti‐replication treatments in the single‐sensitive group. (D) The expression scores of the above altered signatures of the anti‐microtubule treatments and the anti‐replication treatments across the double‐sensitive group, the single‐sensitive group, and the not‐sensitive group (*n* = 42 in the double‐sensitive group, *n* = 54 in the single‐sensitive group, and *n* = 18 in the not‐sensitive group from the anti‐microtubule treatment; *n* = 63 in the double‐sensitive group, *n* = 81 in the single‐sensitive group, and *n* = 27 in the not‐sensitive group from the anti‐ replication treatment). (E) The correlation relationship of the hallmark genesets and the above‐enriched altered signatures.

Then, we directly identified the altered molecular signatures in different combinations of treatments and response groups. The changed signatures of anti‐microtubule treatments in the double‐sensitive group, anti‐replication treatments in the double‐sensitive group, and anti‐microtubule treatments in the single‐sensitive group were coincident with the downregulation of cell division signatures and the upregulation of cell death signatures (Figure 5A,B and Figure S7A). Similarly, the changed signatures of anti‐microtubule treatments in the not‐sensitive group, anti‐replication treatments in the not‐sensitive group, and anti‐replication treatments in the single‐sensitive group were coincident with the downregulation of biosynthesis signatures and the upregulation of cell cycle signatures (Figure 5A,C and Figure S7B). To further explore these four altered signatures, we identified the expression of these signature genes in all the treated PDOs. The upregulated signatures of cell death and cell cycle were highly and specifically expressed and specific in the corresponding response groups (Figure S7C). In the anti‐microtubule treatment group and the anti‐replication group, although these signatures were identified according to comparison with untreated controls, differential expression of these four altered signatures was also in accordance with the corresponding combinations of treatments and response groups (Figure 5D). Through the correlation of hallmark geneset, the downregulated cell division signature and upregulated cell death signature presented the opposite correlation tendencies. The downregulated biosynthesis signature was associated with terms such as epithelial mesenchymal transition, cholesterol homeostasis, apical junction, and complement, while the upregulated cell cycle signature was enriched with immune‐related pathways including inflammatory response, interferon alpha response, and interferon gamma response [38] (Figure 5E). The organoid samples treated by either anti‐microtubule treatments or anti‐replication treatments also presented similar associated hallmark signatures (Figure S7D).

### 2.5 Identification of Response Associated Molecular Signatures and Confirmation of Them in the TCGA Datasets

**FIGURE 6:**
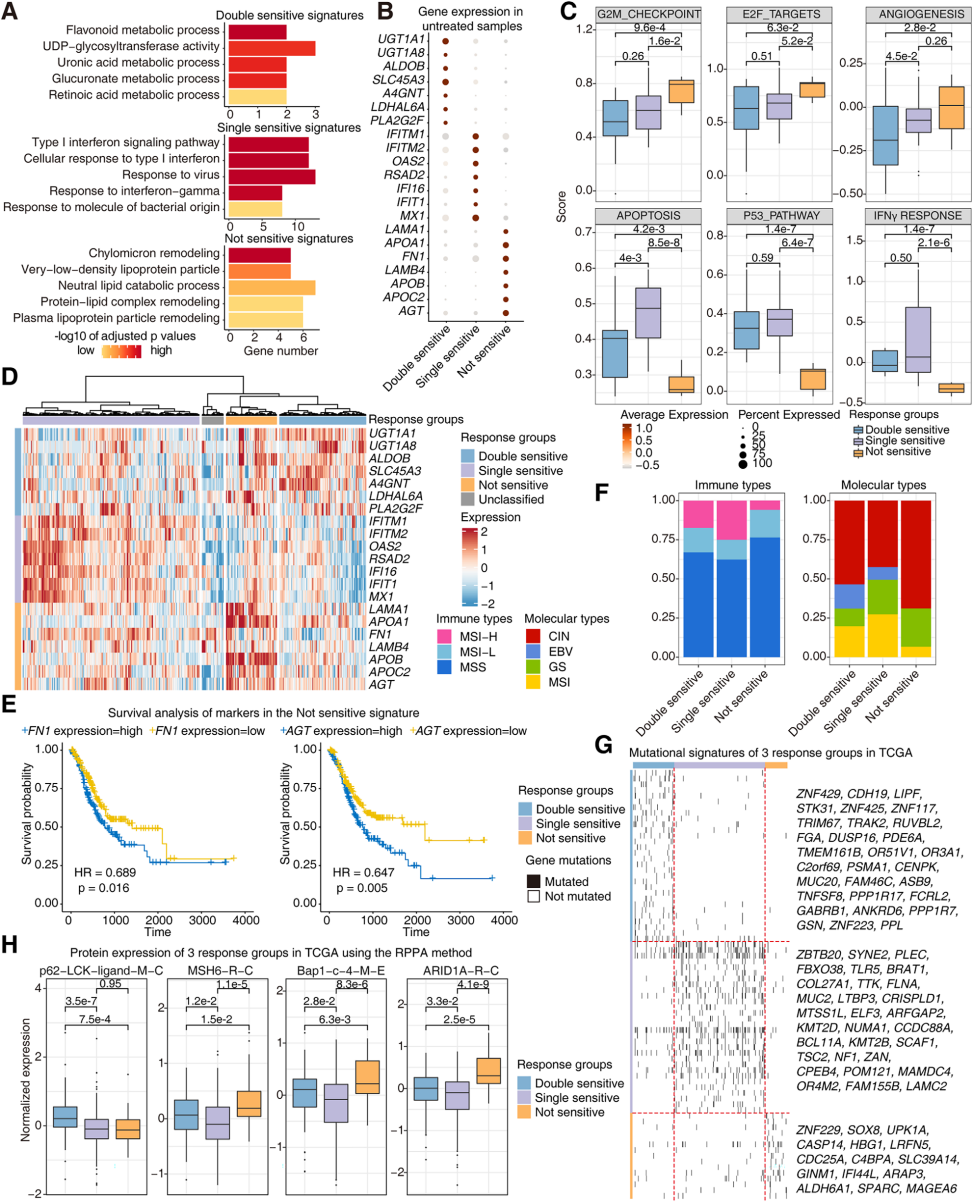
The intrinsic signatures of these three response groups associated with prognosis. (A) The intrinsic features of the three response groups in all the untreated PDOs from this study. (B) Gene expression of the three response groups in all the untreated PDOs from this study. (C) The expression of hallmark genesets in the three response groups of all the untreated PDOs from this study (*n* = 21 in the double‐sensitive group, *n* = 27 in the single‐sensitive group, and *n* = 9 in the not‐sensitive group). (D) The expression of related markers in the identified response groups of GC samples from the TCGA dataset. (E) The survival analysis of *FN1* and *AGT* in all the GC samples from the TCGA dataset. (F) Proportion of immune types and molecular types of the identified response groups of GC samples from the TCGA dataset. (G) Enriched mutational signatures in the identified response groups of GC samples from the TCGA dataset. (H) Protein expression signatures revealed by the RPPA assay in the identified response groups of GC samples from the TCGA dataset (*n* = 116 in the double‐sensitive group, *n* = 236 in the single‐sensitive group, and *n* = 69 in the not‐sensitive group).

The above analyses presented the differentially altered signatures after treatments of three different response groups. Then, we analyzed the transcriptomic information of untreated organoids and the response group classification information to associate the pre‐treatment molecular signatures of response groups with treatment responses. Significantly enriched molecular signatures of untreated organoids among three response groups were presented, with the double‐sensitive group showing glucose metabolism‐related signatures, the single sensitive group showing immune response signatures, and the not‐sensitive group showing lipid metabolism‐related signatures (Figure 6A). Representative markers of corresponding features such as *UGT1A1*, *ALDOB*, *SLC45A3 (for doube‐sensitive group)*, *IFITM1*, *IFI16*, *IFIT1 (for single‐sensitive group)*, *APOA1*, *APOB*, and *APOC2 (for not‐sensitive group)* were expressed specifically in respective response groups (Figure 6B). Furthermore, we scored these hallmark genesets in untreated PDOs of three response groups (Figure 6C and Figure S8A). The not‐sensitive group showed higher activities of G2M checkpoint, E2F targets, angiogenesis, and lower activities of apoptosis, P53 pathway, and interferon alpha/gamma response, which indicated higher malignant features of this group of tumors. Especially, the lipid metabolism‐related signature was reported to be associated with angiogenesis, which implies the potential metastatic tendency of the not‐sensitive samples [39, 40].

To extend our findings to the broader patient group and explore more related characteristics, we applied the above three identified signatures to the GC dataset from TCGA. Excitingly, GC samples in the TCGA dataset were clustered into the three consistent groups by unsupervised clustering of corresponding marker genes (Figure 6D). There were also a few samples without any of the above features, which might be due to the significant intra‐tumor heterogeneities of GCs. With the confirmed classification in the TCGA datasets, we re‐performed the differential analysis and the results showed that the drug response prognosis identification was the same as the signatures in our organoid data, which confirmed the robustness of the classification (Figure S8B,C).

Based on the classification of drug response prediction and multi‐omics datasets of GC samples from TCGA, we explored the multi‐dimensional and integrated signatures of different response groups [4]. As for the association of signature scores and overall survival, though there were no clear relationships between these three signatures and prognosis, higher expressions of the not‐sensitive group markers *FN1* and *AGT* were related to worse prognosis of GC (Figure 6E).

With the clinical information and molecular identification of the cohort, we found that the MSI‐high type and the EBV subtype of GC were essentially depleted in the not‐sensitive group, whereas the CIN subtype of GC was enriched in the not‐sensitive group (Figure 6F). We further investigated the associations between the molecular signatures characterizing our drug response groups and the established molecular subtypes of GC. In the EBV subtype, we identified that high expression of *UGT1A8* and *APOB* within the cohort was significantly associated with worse overall survival, providing potential prognostic markers for the EBV subtype (Figure S8D). In the MSI subtype, survival analysis identified *LDHAL6A*, *FN1*, and *LAMB4* as significant prognostic factors, where high expression of these genes correlated with poor survival outcomes (Figure S8E). Survival analysis in the *TP53*‐mutated samples further confirmed that high expression of *ALDOB* and *FN1* served as significant indicators of poor prognosis in this subset (Figure S8F).

As for immune cell infiltration states, we found that the proportions of macrophages and CD8^+^ T cells were lower in the not‐sensitive group than the other two response groups, while plasma cells had a higher proportion in the not‐sensitive group according to CIBERSORT (Figure S8G). We further analyzed the expression of specific functional gene signatures for plasma cells (including *SDC1, TNFRSF17*, and *SLAMF7*), macrophages (including *MRC1, CSF1R, ARG1, TGFB1, SPP1, TREM2*, and *C1QA*), and exhausted CD8^+^ T cells (including *PDCD1, HAVCR2, LAG3, TIGIT, CXCL13*, and *CTLA4*). Our analysis showed that macrophage markers and exhausted CD8^+^ T‐cell markers showed higher expression in the single‐sensitive group (Figure S9A). We also investigated the prognostic value of these markers within specific response groups. In the single‐sensitive group, high *MRC1* expression was associated with poor prognosis (Figure S9B). Surprisingly, in the not‐sensitive group, high *MRC1* expression correlated with better prognosis (Figure S9C). *MRC1* encodes a transmembrane glycoprotein that serves as a canonical marker for M2‐polarized tumor‐associated macrophages [41, 42]. In addition, high expression level of immunosuppressive markers *CSF1R* and *TGFB1* predicted poor outcomes in the single‐sensitive group. Conversely, high *PDCD1* expression correlated with better prognosis in this group, potentially indicating a reservoir of T cells that could be reinvigorated (Figure S9B). In the not‐sensitive group, high expression of the checkpoint *TIGIT* was associated with better prognosis (Figure S9C).

Finally, we investigated other omics layers in these three response groups. As for the genome alterations, we identified the significantly enriched somatic mutations in these three response groups, respectively (Figure 6G). Several mutated genes in the RAF/MAPK kinase pathway including *FGA*, *PSMA1*, *DUSP16*, *ASB9*, and *PPL* were enriched in the double‐sensitive group, which is explicable to be associated with the altered metabolism signature in this response group. As for the not‐sensitive group, somatic mutations of *CASP14*, *SOX8*, *CDC25A*, and *IFI44L* were enriched, and the mutated state of at least one gene in this mutated geneset was significantly associated with the worse prognosis (Figure 6G and Figure S9D). We also screened the proteomic data of these three response groups using the RPPA assay, and identified the higher protein abundance of p62 LCK ligand in the double‐sensitive group as well as the higher protein abundances of MSH6, BAP1, ARID1A in the not‐sensitive group (Figure 6H). Of them, ARID1A is a chromatin regulatory protein that maintains genome stability and interacts with many oncoproteins and tumor suppressor proteins such as KRAS, PIK3CA, and TP53 (Figure S9E). In summary, the above multi‐dimensional information provided the detailed landscape of these three response groups, which revealed the potential mechanisms and provided more accurate classification criteria.

### 2.6 Verification of Response‐Associated Molecular Signatures and Exploration of Potential Drugs for the Not‐Sensitive Group Based on Public Drug Perturbing Data

**FIGURE 7:**
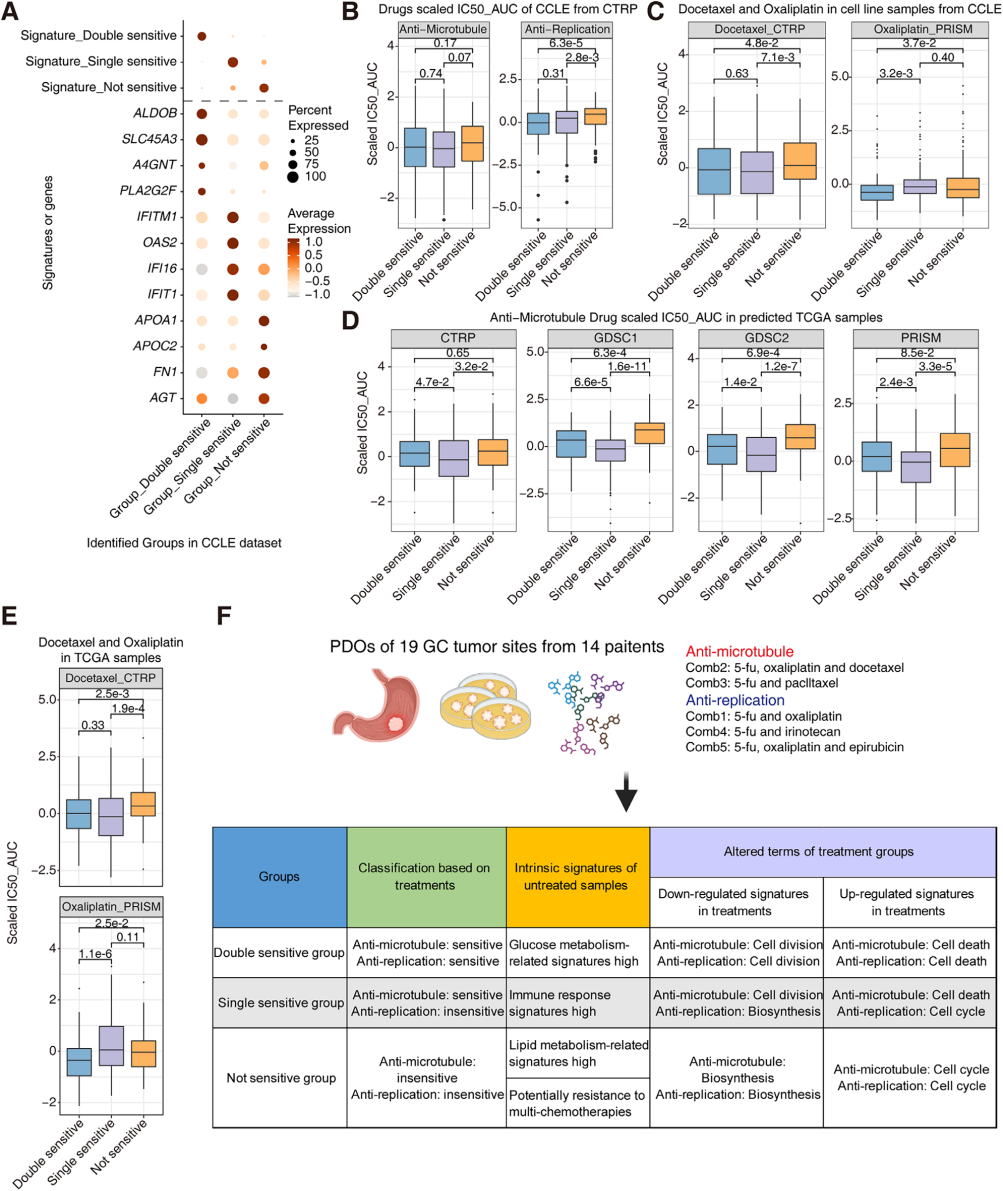
The verification of drug effects for these response groups by CCLE and TCGA datasets. (A) The expression of response‐related genesets and marker genes in the predicted three response groups of the CCLE dataset. (B) The scaled IC50 values of the anti‐microtubule and anti‐replication drugs from the CTRP dataset in the predicted three response groups from the CCLE dataset (*n* = 191 in the double‐sensitive group, *n* = 308 in the single‐sensitive group, and *n* = 222 in the not‐sensitive group). (C) The scaled IC50 values of docetaxel and oxaliplatin from the CTRP and PRISM datasets in the predicted three response groups from the CCLE dataset (*n* = 191 in the double‐sensitive group, *n* = 308 in the single‐sensitive group, and *n* = 222 in the not‐sensitive group). (D) The scaled IC50 values of the anti‐microtubule drugs from the CTRP, GDSC1, GDSC2, and PRISM datasets in the predicted three response groups from the TCGA dataset (*n* = 116 in the double‐sensitive group, *n* = 236 in the single‐sensitive group, and *n* = 69 in the not‐sensitive group). (E) The scaled IC50 values of docetaxel and oxaliplatin from the CTRP and PRISM datasets in the predicted three response groups from the TCGA dataset (*n* = 116 in the double‐sensitive group, *n* = 236 in the single‐sensitive group, and *n* = 69 in the not‐sensitive group). (F) The molecular signatures of identified groups in GC and altered signatures after different chemotherapy drug treatments.

Although the above classification of GC samples provided the potential guidance for precise treatments of GC, external drug response datasets were required to verify drug response effects of these three response groups. First, we used the paired transcriptome data and small‐molecule drug response data of cell lines from the CCLE database, which includes lots of cancer cell lines from different cancer types [43]. Small‐molecule drug response data for the cell lines were from other published datasets [44, 45, 46, 47, 48]. Based on the three response signatures and transcriptome data, we clustered cell line samples into corresponding three groups with prominent signatures, and about half of the cell lines were undefined (Figure S10A). Accordingly, the signature scores and related marker genes were expressed consistently (Figure 7A). Gastrointestinal cancers such as CRC and GC presented the high proportions of the double‐sensitive group, consistent with the common use of these chemotherapy regimens for gastrointestinal cancers (Figure S10B). In these three classified response groups, the IC50 values of anti‐microtubule drugs in the double‐sensitive group and single‐sensitive group were relatively low, and the IC50 values of anti‐replication drugs in the double‐sensitive group were relatively low (Figure 7B). Docetaxel is one of the anti‐microtubule drugs, and oxaliplatin is one of the anti‐replication drugs, which were also used in the chemotherapy regimens in this study. The significantly lower IC50 values of docetaxel were in the double‐sensitive group and single‐sensitive group, and oxaliplatin only presented the low IC50 values in the double‐sensitive group (Figure 7C). Then, we integrated the public transcriptomic data and drug response IC50 values to predict corresponding IC50 values of small‐molecule drugs in GC samples from the TCGA database based on a ridge‐regression model [49, 50]. Combined with the above classifying information, we identified that the anti‐microtubule drugs in the double‐sensitive group and single‐sensitive group across all four datasets had slightly lower IC50 values (Figure 7D). Similarly, docetaxel and oxaliplatin exhibited the corresponding IC50 values based on the classification (Figure 7E). Both these drug responses predicted results of cancer cell lines, and GC samples from TCGA confirmed the clear association of identified molecular signatures and corresponding drug response effects.

## 3 Discussion

GC showed strong variations to current clinical treatments, and there is a lack of molecular classification criteria for different chemotherapy regimens of different GC patients [1, 2]. Here, we developed a robust, convenient and fast system for the evaluation of the clinically first‐line chemotherapy regimens based on PDOs. PDOs from various gastric tumor sites from different patients were classified into three groups based on variant responses to anti‐microtubule and anti‐replication chemotherapies (defined by the specific drugs used in the treatment combination). Treatment perturbation signatures were also identified according to the transcriptome changes, which reflected how GCs responded to different chemotherapy regimens. Moreover, the intrinsic molecular signatures of tumor sites from these three response groups were also identified, which were associated with the prognosis of patients from different subtypes. Finally, we extended these signatures to the GC samples of the TCGA dataset and public drug response datasets, and found that our classification was consistently applicable to all the GC patients (Figure 7F). The main limitation of this investigation lies in the paucity of empirical data to substantiate the current findings.

Using the PDO models, we found clear associations between types of patients and different treatments, especially responses to different treatments of the same patient. Tumors of the patients were classified into three groups: tumors from the double‐sensitive group responded to both the anti‐microtubule and anti‐replication treatments, tumors from the single‐sensitive group only responded to the anti‐microtubule treatments, and tumors from the not‐sensitive group responded to neither the anti‐microtubule nor the anti‐replication treatments. The 1‐month window phase after surgery or pathological diagnosis, the high success rate of organoid establishment, and the rapid expansion culture system allow integrating PDOs into personalized medicine [51, 52].

Based on the classification of response groups, drug perturbation effects were presented. By comparison of the cell viability after treatments (reflecting the good or bad cancer killing effects), we found that protein secretion, heme metabolism, p53 pathway, and tumor differentiation signatures were positively correlated with the good treatment responses, while DNA repair, mitotic spindle, and MYC targets were negatively correlated with the good treatment responses. Meanwhile, these signatures were not absolutely consistent with drug responses. As for tumor differentiation states, P39_T1 and P45_T1 were poorly differentiated and classified as the double sensitive group. Compared with other differentiated samples in the double sensitive group, P39_T1 and P45_T1 exhibited signatures such as carboxylic acid metabolic process, interferon signaling, and small molecular catabolic process (Figure S10C). P19_T1 was well differentiated and classified as the single sensitive group. Compared with other poorly differentiated samples in the single sensitive group, P19_T1 exhibited signatures such as response to xenobiotic stimulus, alanine transport response to reactive oxygen species, and epithelial cell differentiation (Figure S10C).

To further associate the tumor signatures with prognosis of these commonly used chemotherapy regimens, we identified the intrinsic molecular features of untreated tumor organoids from these three response groups. We found that the untreated samples from the double‐sensitive group presented glucose metabolism‐related signatures, the untreated samples from the single‐sensitive group presented immune‐response related signatures, and the untreated samples from the not‐sensitive group presented lipid metabolism‐related signatures. Excitingly, we extended this classification to the GC samples from the TCGA dataset and found that this classification model was effective for all the GC samples. Also, multi‐omics signatures of these three response groups were explored in detail. All the signatures indicated that the not‐sensitive group presented a relatively stable genome. GC samples of the not‐sensitive group from TCGA presented the high expression of *APOA1* and *FN1*, and these genes were found to be associated with neuroendocrine carcinoma (NEC) of GCs in two of our previous cohorts [36, 37].

GC samples of the double‐sensitive group and single‐sensitive group respond well to at least one chemotherapy treatment based on our analyses. Nevertheless, it seemed that the not‐sensitive group does not respond to common chemotherapies of GCs and they may show drug resistance. We identified higher expression of *ABCC2* in the not‐sensitive group from untreated samples in our dataset (Figure S10D). ABCC2 is a member of the superfamily of ATP‐binding cassette (ABC) transporters, and is also known as one of the multidrug resistance‐associated proteins (MRPs). ABC proteins contribute to drug resistance by transporting various molecules outside of a cell across extracellular and intracellular membranes [53, 54]. What is more, we also identified higher expression of ABC family genes in the not‐sensitive group from TCGA, which indicated the universal expression of MRPs in the not‐sensitive group of GCs (Figure S10E). ABCC2 is also associated with several apolipoproteins such as APOB in the protein–protein interaction networks, which also supports the potential drug resistance mechanism (Figure S10F). Therefore, based on these mechanisms, combinations with inhibitors of MRPs or other treatment modalities may benefit the not‐sensitive group of GCs. We screened this dataset for drugs that are functionally characterized as MRP inhibitors of ABC transporter activity, including ibrutinib, ketoprofen, naproxen, omeprazole, and rabeprazole [55, 56, 57]. We analyzed the predicted IC50 values of these five drugs across our three defined response groups (Figure S10G). Strikingly, we observed that the not‐sensitive group—which we previously characterized as having high *ABCC2* expression and multidrug resistance features—exhibited significantly lower IC50 values for these specific drugs compared to the double‐sensitive and single‐sensitive groups.

In summary, our study provided a robust and convenient system for GC chemotherapy regimen selection, which can also be applied to other types of cancer. With this system, we analyzed multifaceted signatures of GC chemotherapy regimens and linked them to corresponding treatment responses. Our study deepens the understanding of first‐line chemotherapy regimens and provides a rich resource for guiding optimal selection of clinical treatments of GC patients.

## 4 Materials and Methods

### 4.1 PDO Culture

The cell precipitates were resuspended in Matrigel (356231, Corning) with a volume ratio of 1:5. Cells were seeded in a 24‐well plate at 60 µL drops per well. After incubating the plate at 37°C for 15 min to solidify the Matrigel, 500 µL COM was added to each well and refreshed every 3 days. For the first 3 days, 10 µM Y‐27632 (S1049, Selleckchem) should be added to the medium. TrypLE (12604‐021, Gibco) was used for passage. In brief, COM was discarded and 500 µL TrypLE was added to each well. The solidified Matrigel was pipetted and collected into a tube, then it was digested at 37°C for 5 min. Cells were spun down and resuspended for subculture according to abovementioned steps. Freezing and thawing were consistent with conventional methods.

### 4.2 Drug Response Assay

We took six chemotherapy drugs and first analyzed their IC50 values for PDOs. Note that 2500–5000 cells were seeded in a 96‐well plate at 8 µL drops per well. Cell viability was detected using the CellTiter‐Glo 3D Reagent (G9683, Promega) according to the manufacturer's instructions on Day 5 after drug exposure to reflect the cellular response to drugs. The results were normalized to control and analyzed by GraphPad Prism 6 to get IC50 values. The median IC50 values for 5‐fluorouracil, docetaxel, epirubicin, irinotecan, oxaliplatin, and paclitaxel were 2.348 µM, 16.94 nM, 100.4 nM, 6.729 µM, 22.55 µM, and 10.49 nM, respectively. Further detailed methods can be found in the Supporting Information.

## Author Contributions

X.Z., F.T., and W.F. supervised the work. F.T., X.Z., J.Y., S.Q., Y.G., and J.L. conceived the idea. S.Q. and Y.G. performed the sample preparation and experiments. X.Z. acquired the human tissue samples and performed pathology reviews. J.Y. and J.L. performed the bioinformatics analyses. J.Y., S.Q., and F.T. wrote the manuscript. L.D., X.W., Y.Z., Y.L., Y.M., J.S., L.X., and L.W. participated in the method section writing, review, and editing of the manuscript with the help of all authors. All authors have read and approved the final manuscript.

## Ethics Statement

This study was reviewed and approved by the Ethics Committee of Peking University Third Hospital (License No. IRB00006761‐M2016170). All procedures were undertaken in accordance with the Helsinki Declaration. Informed consents were obtained from all enrolled patients.

## Conflicts of Interest

The authors declare no conflicts of interest.

## Supporting information




**Supporting File 1**: mco270656‐sup‐0001‐SuppMat.pdf

**Supporting File 2**: mco270656‐sup‐0002‐TableS1.xlsx

**Supporting File 3**: mco270656‐sup‐0003‐TableS2.xlsx

**Supporting File 4**: mco270656‐sup‐0004‐TableS3.xlsx

**Supporting File 5**: mco270656‐sup‐0005‐TableS4.xlsx

## Data Availability

The raw sequencing data in this paper have been deposited in the Genome Sequence Archive with accession number HRA006702.
