# Supplementary material for: Systematic Identification of Molecular Signatures Dictating Therapeutic Effects of Clinically First‐Line Chemotherapy Regimens for Human Gastric Cancer Patients Based on Organoid Model
Source: MedComm (2020). 2026 Mar 2;7(3):e70656. doi: 10.1002/mco2.70656 (PMC12954136; doi:10.1002/mco2.70656)
Supplement: Supplementary file 1 — Supporting File 1: mco270656‐sup‐0001‐SuppMat.pdf [file MCO2-7-e70656-s003.pdf]

Supplementary Information

Systematic identification of molecular signatures dictating therapeutic effects of clinically first-line chemotherapy regimens for human gastric cancer patients based on organoid model

Supplementary Methods

Patient tissues dissociation and organoid culture

Human GC tissues were collected from surgically resected GC specimens. Tissues were rinsed with Dulbecco's phosphate buffered saline (DPBS, D8537, Sigma-Aldrich), then they were minced into less than 3 mm^3 pieces and digested with 1.5 mg/mL collagenase type II (17101-015, Gibco) and 1.5 mg/mL type IV (17104-019, Gibco) in advanced DMEM/F12 medium (12630-010, Gibco). Tissue pieces were incubated at 37℃ and pipetted occasionally until they disappeared. After filtering through a 70 μm strainer, dissociated cells were centrifuged for 5 min at 500 g and washed with advanced DMEM/F12 medium twice.

Culture medium composition

The conditioned medium (COM) was used to culture organoids. It was 1× N-2-hydroxyethylpiperazine-N-2-ethane sulfonic acid (HEPES; 15630-080, Gibco), 1× GlutaMax (35050-061, Gibco), 100 units/mL penicillin, 100 μg/mL streptomycin (15140-122, Gibco),1 mg/mL primocin (#anti-pm, InvivoGen), 1 mM N-acetylcysteine (A9165, Sigma-Aldrich), 1× N2 supplement (17502-048, Gibco), 1× B27 supplement (17504-044, Gibco), 10 mM nicotinamide (N0636, Sigma-Aldrich), 50 ng/mL epidermal growth factor (EGF, #AF-100-15, PeproTech), 200 ng/mL fibroblast growth factor 10 (FGF10, #100-26, PeprroTech), 2 μM A83-01 (2939, Tocris Bioscience), and 1 nM gastrin (G9145, Sigma-Aldrich) in advanced DMEM/F12 medium with 50% R-spondin 3, Wnt3A, and Noggin conditioned medium, which generated from L-WRN cells (CRL-3276, ATCC).

H&E staining

Sample were fixed with 4% paraformaldehyde (P1110, Macgene) and then embedded in paraffin for slices preparation. Slides were dewaxed with xylene, ethanol and water stepwise. Then we disseminated slides into hematoxylin for about 10 min. After flushing out the floating color with hydrochloric acid alcohol and water, slides were moved into eosin for about 3 min. Finally, slides were dehydrated and made to be transparent with ethanol and xylene, and sealed carefully.

Drug combination screening

Drug combinations were selected based on clinical practice guidelines and primary literature. We set the 5-fluorouracil as control because all combinations contained it. Other drug concentrations normalized to 5-fluorouracil according to plasma concentrations. For example, the concentration of docetaxel was plasma concentration of docetaxel/ plasma concentration of 5-fluorouracil* IC50 value of 5-fluorouracil. Other drug concentrations were calculated similarly. Plasma concentrations were referred to literature[1–7]. In this way, the final used concentrations for drugs in different combinations are: 5-fluorouracil - 2 μM; oxaliplatin - 4 μM; docetaxel - 10 nM; paclitaxel - 10 nM; irinotecan - 25 nM; epirubicin - 2 nM. Then PDOs were digested and seeded in a 96-well plate as abovementioned. Each drug combination treatment group had 4 biological replicates. 20,000–40,000 cells were seeded in a 12-well plate at a 30 μL drop. For initial 3 days, PDOs were culture with COM, and drugs were added on the third day. 3 days later, culture mediums with drugs were refreshed. PDOs were treated with drugs for 5 days in total. The cells in 96-well plates were used for cell viability assay using the CellTiter-Glo 3D Reagent and cells in 12-cell plates were used for bulk RNA sequencing.

Bulk RNA sequencing

Bulk transcriptome sequencing libraries were constructed according to previously published methods with some modifications[8]. PDOs were dissociated into single cells and stained with 7-AAD viability staining solution (Biolegend, 420403) for fluorescence-activated cell sorting (FACS, BD FACS ARIA SORP, BD Biosciences) on the fifth day of drug treatment. And 100 7-AAD$^-$ cells were sorted into strip tubes with 8 μL lysis buffer (1 U/μL RNase Inhibitor (2313B, TAKARA), 2.5 mM dNTP (4019, TAKARA), 0.75 μM oligo dT primer, and 0.475% Triton X-100 (T8787, Sigma-Aldrich) in nuclease-free water). Each drug combination regimen had three replicates. We used SuperScript II reverse transcriptase (18064071, Invitrogen), template switch oligo primer, and barcoded primers with unique molecular identifiers (UMIs) to perform reverse transcription. And then cDNAs were amplified by KAPK HiFi HotStart ReadyMix (KK8504, KAPA Biosystems) and purified by Ampure XP beads (A63882, Beckman). The libraries were constructed by TruePrep DNA Library Prep Kit V2 for Illumina (TD502-02, Vazyme) according to the manufacturer's instructions with half of the recommended volume. The library sequencing was performed on the Illumina NovaSeq 6000 platform on the paired-end 150-bp mode.

Processing of bulk RNA-seq data

We used fastp (version 0.23.1) to trim reads of low quality or with adaptors and used STAR (version 2.7.0f) to map reads to hg38 reference genome. FeatureCounts (version 2.0.1) was used to calculate counts of every gene. The R package DESeq2 (version 1.24.0) was used to identify differential expressed genes (DEGs), and the gene count matrix was inputted. The adjusted p value was calculated by the Benjamini-Hochberg (BH) method, and the cut-off was 0.05. The dimensionality reduction analyses including PCA and uniform manifold approximation and projection (UMAP) were performed using the calculating frame of the R package Seurat (version 3.2.3), and the function “AddModuleScore” was used to calculate scores of specific genesets.

Geneset enrichment analysis

The GO analysis was performed by clusterProfiler (version 3.18.0) or the online tool Metascape (https://metascape.org/). We also used the GSVA (Gene Set Variation Analysis) enrichment scores to describe enrichment scores of genesets and signatures through the R package GSVA (version 1.32.0). The function “gsva” of the GSVA package was performed and the “ssgsea” method was used. The parameters were “min.sz = 1, max.sz = Inf”.

Identification of drug groups and sample groups

As for anti-microtubule, anti-replication drug groups and double-sensitive, single-sensitive and not-sensitive sample groups, the mean values of cell viability were used, and the hierarchical clustering method (using the “ward” method) was used to identify the drug level and sample level groups. As for clustering of samples with altered molecular signatures comparing with control, the log of P values of enriched up- and down-regulated terms were inputted, and the hierarchical clustering method was also used to cluster samples.

Published datasets and geneset resources

A study about drug screening based on CRC organoids was used[9]. The drug information, cell

viability values and GR50 values were also used. The transcriptome data and drug response data of CCLE, as well as multi-omics data of TCGA were used and downloaded from corresponding data portal, respectively.
As for used genesets, hallmark genesets were from the MSigDB database (https://www.gsea-msigdb.org/gsea/msigdb). Tumor differentiation genesets were from three individual studies[10–12]. Cell death pathways (including apoptosis, ferroptosis and pyroptosis) related genesets were sourced from ontology gene sets from the MSigDB database and other summarized genesets, with detailed genes in Supplementary Table 3.

The survival analysis
Survival analysis of GC samples from the TCGA dataset based on the expression status of identified genes was carried out by the survival package (version 0.4.8) and the survminer package (version 2.44-1). The assumption of the Cox proportional hazards model was tested using the cox with 0.1 as the cutoff value, and the Cox proportional hazards model was fit using patient groups divided by the median gene expression level.

Analysis of multi-omics data in GC samples from TCGA
Multi-omics data of GC samples from TCGA was downloaded from the data portal of UCSC Xena (https://xenabrowser.net/). The download date is in February 2024. Based on the identified molecular signatures associated with treatment responses, GC samples from TCGA were clustered by hierarchical clustering. Based on the clustering information, differential analyses of multi-omics data were performed. GC sample molecular classifying information was annotated by a previous study. Somatic mutations enriched in respective groups were identified by the Fisher test with a P value less than 0.05 and odd ratio more than 1. DEGs of respective groups were identified by the default pipeline of the R package edgeR (version 3.32.0), with the log_2 value of fold change more than 1.5 and adjusted P values less than 0.05. The differential protein expression in respective groups was identified using the pairwise wilcox test, with the P value less than 0.05. Immune cell infiltration information of GC samples was from one previous TCGA pan-cancer analysis and calculated by the web tool CIBERSORTx[13] (https://cibersortx.stanford.edu/).

Drug response prediction
Based on the transcriptome data of CCLE and TCGA as well as drug response values of small-molecule drugs, a ridge-regression model of the R package pRRophetic (version 0.5) was used to predict drug response values in GC samples from TCGA[14,15]. The predicted AUC values were scaled in the downstream analysis. The differential comparison of scaled AUC values in different groups was performed by the wilcox test.

References

1. Yang RX, Ren HX, Zhuang L, et al. Pharmacokinetic and Myocardial Enzyme Profiles of Two Administration Routes of Epirubicin in Breast Cancer Patients. *Arzneimittelforschung*. 2012;62(12):677-681.
2. Diasio RB, Lakings DE, Bennett JE. Evidence for Conversion of 5-Fluorocytosine to 5-Fluorouracil in Humans: Possible Factor in 5-Fluorocytosine Clinical Toxicity. *Antimicrob Agents Chemother*. 1978;14(6):903-908.
3. Hertz DL, Kidwell KM, Vangipuram K, et al. Paclitaxel Plasma Concentration after the First Infusion Predicts Treatment-Limiting Peripheral Neuropathy. *Clinical Cancer Research*. 2018;24(15):3602-3610.
4. Kenmotsu H, Tanigawara Y. Pharmacokinetics, dynamics and toxicity of docetaxel: Why the Japanese dose differs from the Western dose. *Cancer Science*. 2015;106(5):497-504.
5. Ehrsson H, Wallin I, Yachnin J. Pharmacokinetics of Oxaliplatin in Humans. *MO*. 2002;19(4):261-266.
6. Lurvink RJ, Tajzai R, Rovers KP, et al. Systemic Pharmacokinetics of Oxaliplatin After Intraperitoneal Administration by Electrostatic Pressurized Intraperitoneal Aerosol Chemotherapy (ePIPAC) in Patients with Unresectable Colorectal Peritoneal Metastases in the CRC-PIPAC Trial. *Ann Surg Oncol*. 2021;28(1):265-272.
7. Chabot GG. Clinical Pharmacokinetics of Irinotecan: *Clinical Pharmacokinetics*. 1997;33(4):245-259.
8. Gao Y, Zhang J, Liu Z, et al. Single-cell Sequencing Reveals Clearance of Blastula Chromosomal Mosaicism in In Vitro Fertilization Babies. *Genomics, Proteomics & Bioinformatics*. 2022;20(6):1224-1231.
9. Mao Y, Wang W, Yang J, et al. Drug repurposing screening and mechanism analysis based on human colorectal cancer organoids. *Protein & Cell*. 2024;15(4):285-304.
10. Zhang M, Hu S, Min M, et al. Dissecting transcriptional heterogeneity in primary gastric adenocarcinoma by single cell RNA sequencing. *Gut*. 2021;70(3):464-475.
11. Zhou X, Yang J, Lu Y, et al. Relationships of tumor differentiation and immune infiltration in gastric cancers revealed by single-cell RNA-seq analyses. *Cell Mol Life Sci*. 2023;80(2):57.
12. Bian S, Wang Y, Zhou Y, et al. Integrative single-cell multiomics analyses dissect molecular signatures of intratumoral heterogeneities and differentiation states of human gastric cancer. *National Science Review*. 2023;10(6):nwad094.
13. Newman AM, Steen CB, Liu CL, et al. Determining cell type abundance and expression from bulk tissues with digital cytometry. *Nat Biotechnol*. 2019;37(7):773-782.
14. Geeleher P, Cox NJ, Huang RS. Clinical drug response can be predicted using baseline gene expression levels and in vitro drug sensitivity in cell lines. *Genome Biol*. 2014;15(3):R47.
15. Maeser D, Gruener RF, Huang RS. oncoPredict: an R package for predicting *in vivo* or cancer patient drug response and biomarkers from cell line screening data. *Briefings in Bioinformatics*. 2021;22(6):bbab260.

Figure S1

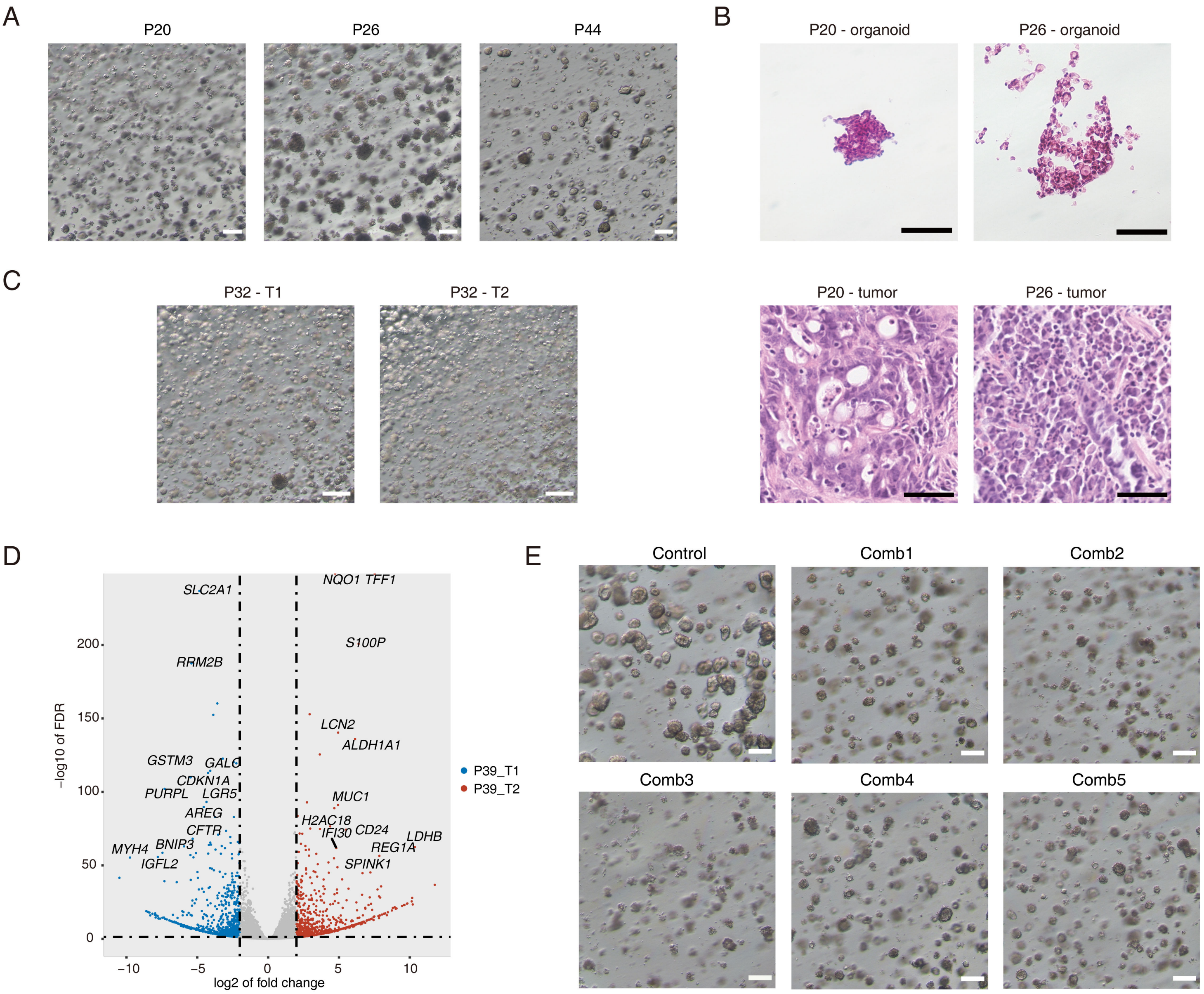
A
P20
P26
P44
B
P20 - organoid
P26 - organoid
P20 - tumor
P26 - tumor
C
P32 - T1
P32 - T2
D
SLC2A1
RRM2B
GSTM3
GALC
CDKN1A
PURPL
LGR5
AREG
CFTR
BNIP3
MYH4
IGFL2
NQO1 TFF1
S100P
LCN2
ALDH1A1
MUC1
H2AC18
IFI30
CD24
LDHB
REG1A
SPINK1
P39_T1
P39_T2
−log10 of FDR
log2 of fold change
0
50
100
150
200
−10
−5
0
5
10
E
Control
Comb1
Comb2
Comb3
Comb4
Comb5

Figure S1. Imaging and HE staining of GC organoids

A. The bright field images of tumor organoids from P20, P26, and P44. Scale bar: 200 μm.
B. H&E staining of the primary tumors and PDOs from P20 and P26. Scale bar: 100 μm for tumor organoids and 50 μm for primary tumors.
C. The bright field images of PDOs from P32_T1 and P32_T2. Scale bar: 300 μm.
D. The volcano plot shows the DEGs of P39_T1 and P39_T2.
E. The bright field images of PDOs after treatments. Scale bar: 200 μm.

Figure S2

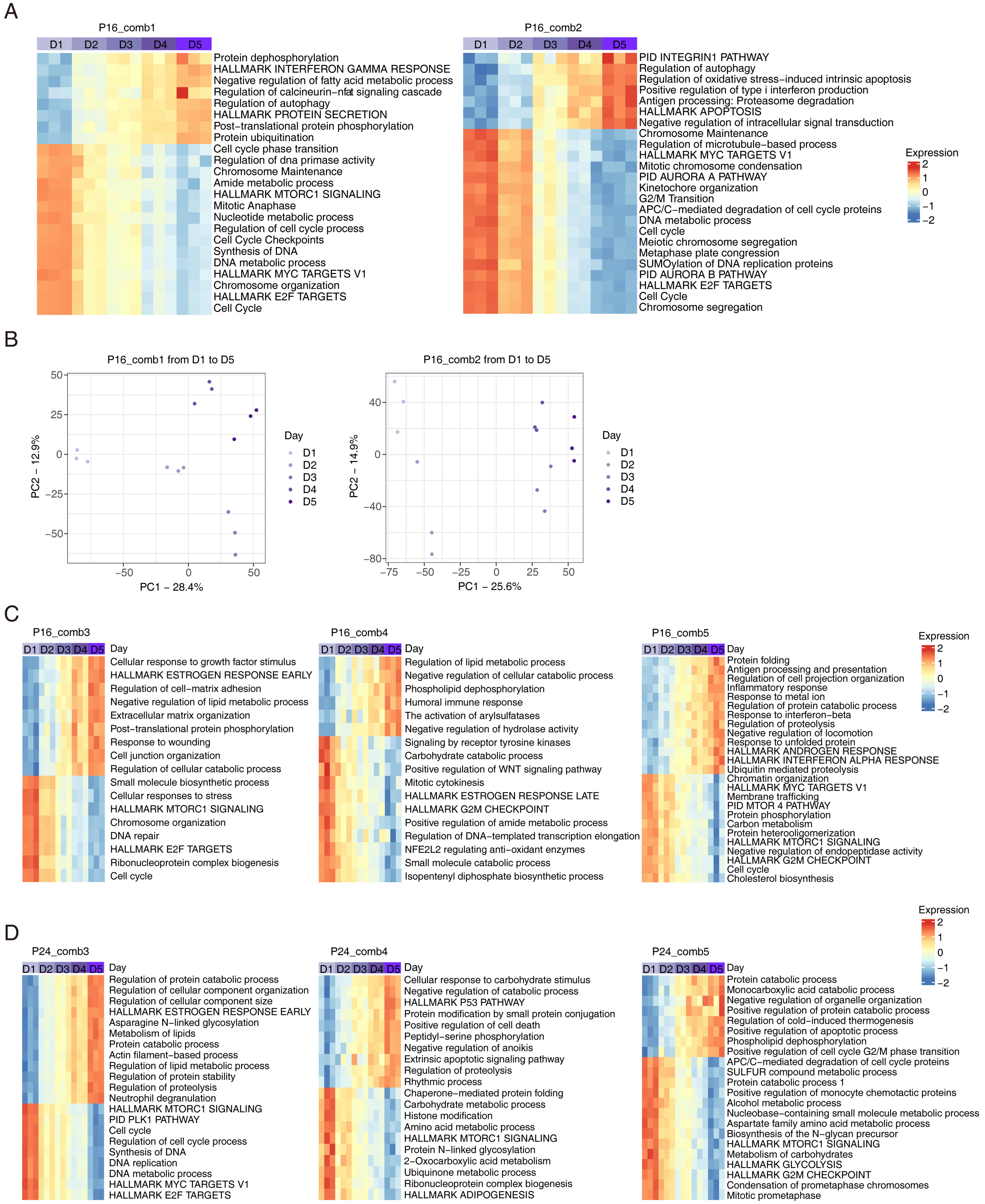
A
P16_comb1
D1 D2 D3 D4 D5
Protein dephosphorylation
HALLMARK INTERFERON GAMMA RESPONSE
Negative regulation of fatty acid metabolic process
Regulation of calcineurin−nfat signaling cascade
Regulation of autophagy
HALLMARK PROTEIN SECRETION
Post−translational protein phosphorylation
Protein ubiquitination
Cell cycle phase transition
Regulation of dna primase activity
Chromosome Maintenance
Amide metabolic process
HALLMARK MTORC1 SIGNALING
Mitotic Anaphase
Nucleotide metabolic process
Regulation of cell cycle process
Cell Cycle Checkpoints
Synthesis of DNA
DNA metabolic process
HALLMARK MYC TARGETS V1
Chromosome organization
HALLMARK E2F TARGETS
Cell Cycle
P16_comb2
D1 D2 D3 D4 D5
PID INTEGRIN1 PATHWAY
Regulation of autophagy
Regulation of oxidative stress−induced intrinsic apoptosis
Positive regulation of type i interferon production
Antigen processing: Proteasome degradation
HALLMARK APOPTOSIS
Negative regulation of intracellular signal transduction
Chromosome Maintenance
Regulation of microtubule−based process
HALLMARK MYC TARGETS V1
Mitotic chromosome condensation
PID AURORA A PATHWAY
Kinetochore organization
G2/M Transition
APC/C−mediated degradation of cell cycle proteins
DNA metabolic process
Cell cycle
Meiotic chromosome segregation
Metaphase plate congression
SUMOylation of DNA replication proteins
PID AURORA B PATHWAY
HALLMARK E2F TARGETS
Cell Cycle
Chromosome segregation
Expression
2
1
0
−1
−2
B
P16_comb1 from D1 to D5
PC1 − 28.4%
PC2 − 12.9%
50
25
0
−25
−50
−50
0
50
Day
D1
D2
D3
D4
D5
P16_comb2 from D1 to D5
PC1 − 25.6%
PC2 − 14.9%
40
0
−40
−80
−75
−50
−25
0
25
50
Day
D1
D2
D3
D4
D5
C
P16_comb3
D1 D2 D3 D4 D5 Day
Cellular response to growth factor stimulus
HALLMARK ESTROGEN RESPONSE EARLY
Regulation of cell−matrix adhesion
Negative regulation of lipid metabolic process
Extracellular matrix organization
Post−translational protein phosphorylation
Response to wounding
Cell junction organization
Regulation of cellular catabolic process
Small molecule biosynthetic process
Cellular responses to stress
HALLMARK MTORC1 SIGNALING
Chromosome organization
DNA repair
HALLMARK E2F TARGETS
Ribonucleoprotein complex biogenesis
Cell cycle
P16_comb4
D1 D2 D3 D4 D5 Day
Regulation of lipid metabolic process
Negative regulation of cellular catabolic process
Phospholipid dephosphorylation
Humoral immune response
The activation of arylsulfatases
Negative regulation of hydrolase activity
Signaling by receptor tyrosine kinases
Carbohydrate catabolic process
Positive regulation of WNT signaling pathway
Mitotic cytokinesis
HALLMARK ESTROGEN RESPONSE LATE
HALLMARK G2M CHECKPOINT
Positive regulation of amide metabolic process
Regulation of DNA−templated transcription elongation
NFE2L2 regulating anti−oxidant enzymes
Small molecule catabolic process
Isopentenyl diphosphate biosynthetic process
P16_comb5
D1 D2 D3 D4 D5 Day
Protein folding
Antigen processing and presentation
Regulation of cell projection organization
Inflammatory response
Response to metal ion
Regulation of protein catabolic process
Response to interferon−beta
Regulation of proteolysis
Negative regulation of locomotion
Response to unfolded protein
HALLMARK ANDROGEN RESPONSE
HALLMARK INTERFERON ALPHA RESPONSE
Ubiquitin mediated proteolysis
Chromatin organization
HALLMARK MYC TARGETS V1
Membrane trafficking
PID MTOR 4 PATHWAY
Protein phosphorylation
Carbon metabolism
Protein heterooligomerization
HALLMARK MTORC1 SIGNALING
Negative regulation of endopeptidase activity
HALLMARK G2M CHECKPOINT
Cell cycle
Cholesterol biosynthesis
Expression
2
1
0
−1
−2
D
P24_comb3
D1 D2 D3 D4 D5 Day
Regulation of protein catabolic process
Regulation of cellular component organization
Regulation of cellular component size
HALLMARK ESTROGEN RESPONSE EARLY
Asparagine N−linked glycosylation
Metabolism of lipids
Protein catabolic process
Actin filament−based process
Regulation of lipid metabolic process
Regulation of protein stability
Regulation of proteolysis
Neutrophil degranulation
HALLMARK MTORC1 SIGNALING
PID PLK1 PATHWAY
Cell cycle
Regulation of cell cycle process
Synthesis of DNA
DNA replication
DNA metabolic process
HALLMARK MYC TARGETS V1
HALLMARK E2F TARGETS
P24_comb4
D1 D2 D3 D4 D5 Day
Cellular response to carbohydrate stimulus
Negative regulation of catabolic process
HALLMARK P53 PATHWAY
Protein modification by small protein conjugation
Positive regulation of cell death
Peptidyl−serine phosphorylation
Negative regulation of anoikis
Extrinsic apoptotic signaling pathway
Regulation of proteolysis
Rhythmic process
Chaperone−mediated protein folding
Carbohydrate metabolic process
Histone modification
Amino acid metabolic process
HALLMARK MTORC1 SIGNALING
Protein N−linked glycosylation
2−Oxocarboxylic acid metabolism
Ubiquinone metabolic process
Ribonucleoprotein complex biogenesis
HALLMARK ADIPOGENESIS
P24_comb5
D1 D2 D3 D4 D5 Day
Protein catabolic process
Monocarboxylic acid catabolic process
Negative regulation of organelle organization
Positive regulation of protein catabolic process
Regulation of cold−induced thermogenesis
Positive regulation of apoptotic process
Phospholipid dephosphorylation
Positive regulation of cell cycle G2/M phase transition
APC/C−mediated degradation of cell cycle proteins
SULFUR compound metabolic process
Protein catabolic process 1
Positive regulation of monocyte chemotactic proteins
Alcohol metabolic process
Nucleobase−containing small molecule metabolic process
Aspartate family amino acid metabolic process
Biosynthesis of the N−glycan precursor
HALLMARK MTORC1 SIGNALING
Metabolism of carbohydrates
HALLMARK GLYCOLYSIS
HALLMARK G2M CHECKPOINT
Condensation of prometaphase chromosomes
Mitotic prometaphase
Expression
2
1
0
−1
−2

Figure S2. Signatures associated with cell viabilities after treatments.

A. Differential signatures expression during the treatment time from day 1 to day 5 of Comb1 and Comb2 for P16. D, day.
B. PCA from day 1 to day 5 of Comb1 and Comb2 for P16. D, day.
C. Differential signatures expression during the treatment time from day1 to day5 of Comb3, Comb4, and Comb5 for P16. D, day.
D. Differential signatures expression during the treatment time from day1 to day5 of Comb3, Comb4, and Comb5 for P24. D, day.

Figure S3

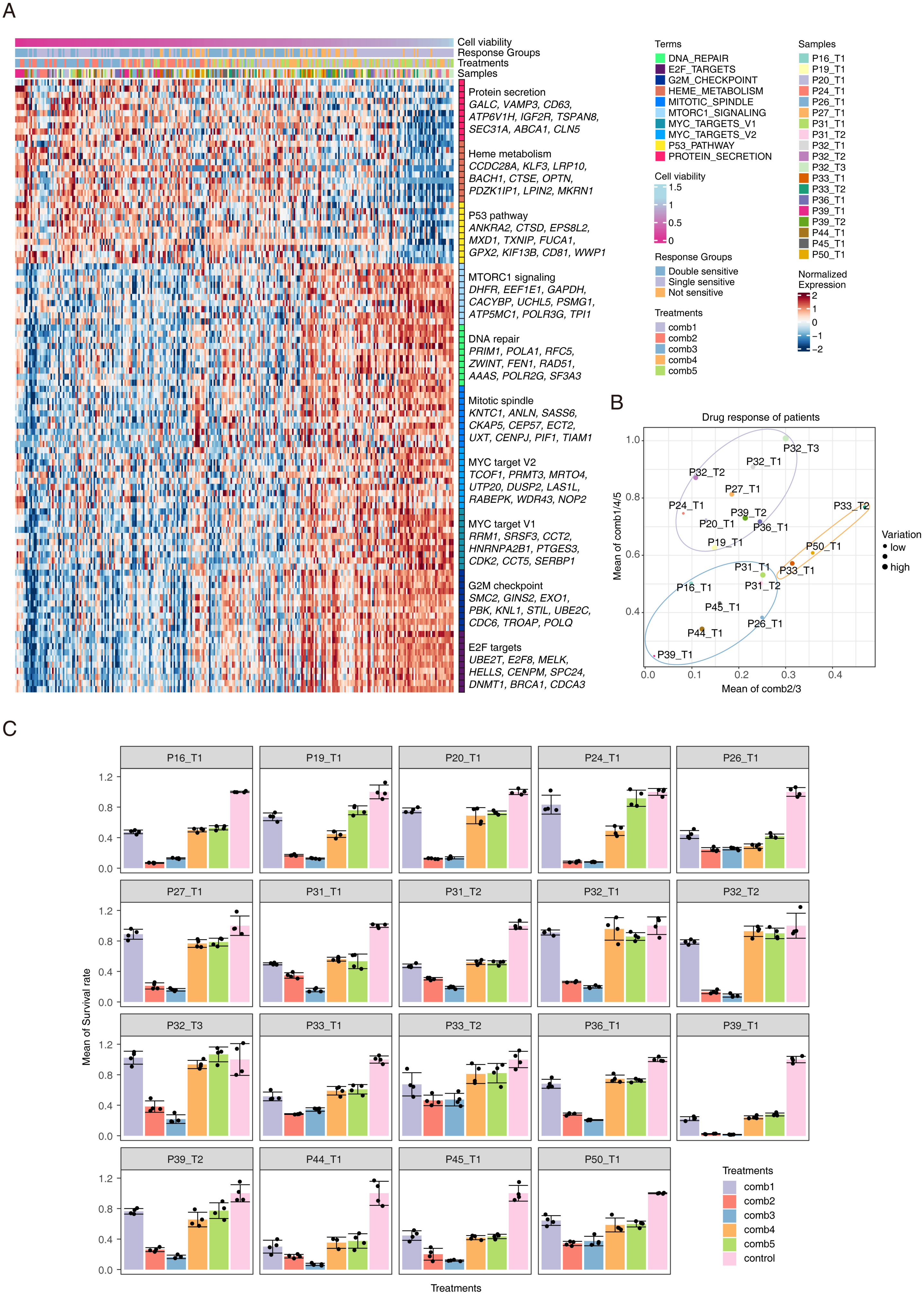

A
Cell viability
Response Groups
Treatments
Samples
Protein secretion
GALC, VAMP3, CD63, ATP6V1H, IGF2R, TSPAN8, SEC31A, ABCA1, CLN5
Heme metabolism
CCDC28A, KLF3, LRP10, BACH1, CTSE, OPTN, PDZK1IP1, LPIN2, MKRN1
P53 pathway
ANKRA2, CTSD, EPS8L2, MXD1, TXNIP, FUCA1, GPX2, KIF13B, CD81, WWP1
MTORC1 signaling
DHFR, EEF1E1, GAPDH, CACYBP, UCHL5, PSMG1, ATP5MC1, POLR3G, TPI1
DNA repair
PRIM1, POLA1, RFC5, ZWINT, FEN1, RAD51, AAAS, POLR2G, SF3A3
Mitotic spindle
KNTC1, ANLN, SASS6, CKAP5, CEP57, ECT2, UXT, CENPJ, PIF1, TIAM1
MYC target V2
TCOF1, PRMT3, MRTO4, UTP20, DUSP2, LAS1L, RABEPK, WDR43, NOP2
MYC target V1
RRM1, SRSF3, CCT2, HNRNPA2B1, PTGES3, CDK2, CCT5, SERBP1
G2M checkpoint
SMC2, GINS2, EXO1, PBK, KNL1, STIL, UBE2C, CDC6, TROAP, POLQ
E2F targets
UBE2T, E2F8, MELK, HELLS, CENPM, SPC24, DNMT1, BRCA1, CDCA3
Terms
DNA_REPAIR
E2F_TARGETS
G2M_CHECKPOINT
HEME_METABOLISM
MITOTIC_SPINDLE
MTORC1_SIGNALING
MYC_TARGETS_V1
MYC_TARGETS_V2
P53_PATHWAY
PROTEIN_SECRETION
Cell viability
1.5
1
0.5
0
Response Groups
Double sensitive
Single sensitive
Not sensitive
Treatments
comb1
comb2
comb3
comb4
comb5
Samples
P16_T1
P19_T1
P20_T1
P24_T1
P26_T1
P27_T1
P31_T1
P31_T2
P32_T1
P32_T2
P32_T3
P33_T1
P33_T2
P36_T1
P39_T1
P39_T2
P44_T1
P45_T1
P50_T1
Normalized Expression
2
1
0
−1
−2
B
Drug response of patients
Mean of comb1/4/5
1.0
0.8
0.6
0.4
Mean of comb2/3
0.0
0.1
0.2
0.3
0.4
P32_T3
P32_T1
P32_T2
P27_T1
P24_T1
P33_T2
P39_T2
P20_T1
P36_T1
P19_T1
P50_T1
P31_T1
P33_T1
P16_T1
P31_T2
P45_T1
P26_T1
P44_T1
P39_T1
Variation
low
high
C
Mean of Survival rate
Treatments
P16_T1
P19_T1
P20_T1
P24_T1
P26_T1
P27_T1
P31_T1
P31_T2
P32_T1
P32_T2
P32_T3
P33_T1
P33_T2
P36_T1
P39_T1
P39_T2
P44_T1
P45_T1
P50_T1
1.2
0.8
0.4
0.0
Treatments
comb1
comb2
comb3
comb4
comb5
control

Figure S3. Signatures associated with cell viabilities after treatments.

A. Gene expression of detailed genes from several genesets arranged by the cell viabilities.
B. The dot plot shows the distribution of different PDOs arranged by the mean of Comb2/3 and Comb1/4/5.
C. Mean cell viabilities of PDOs after different treatments.

Figure S4

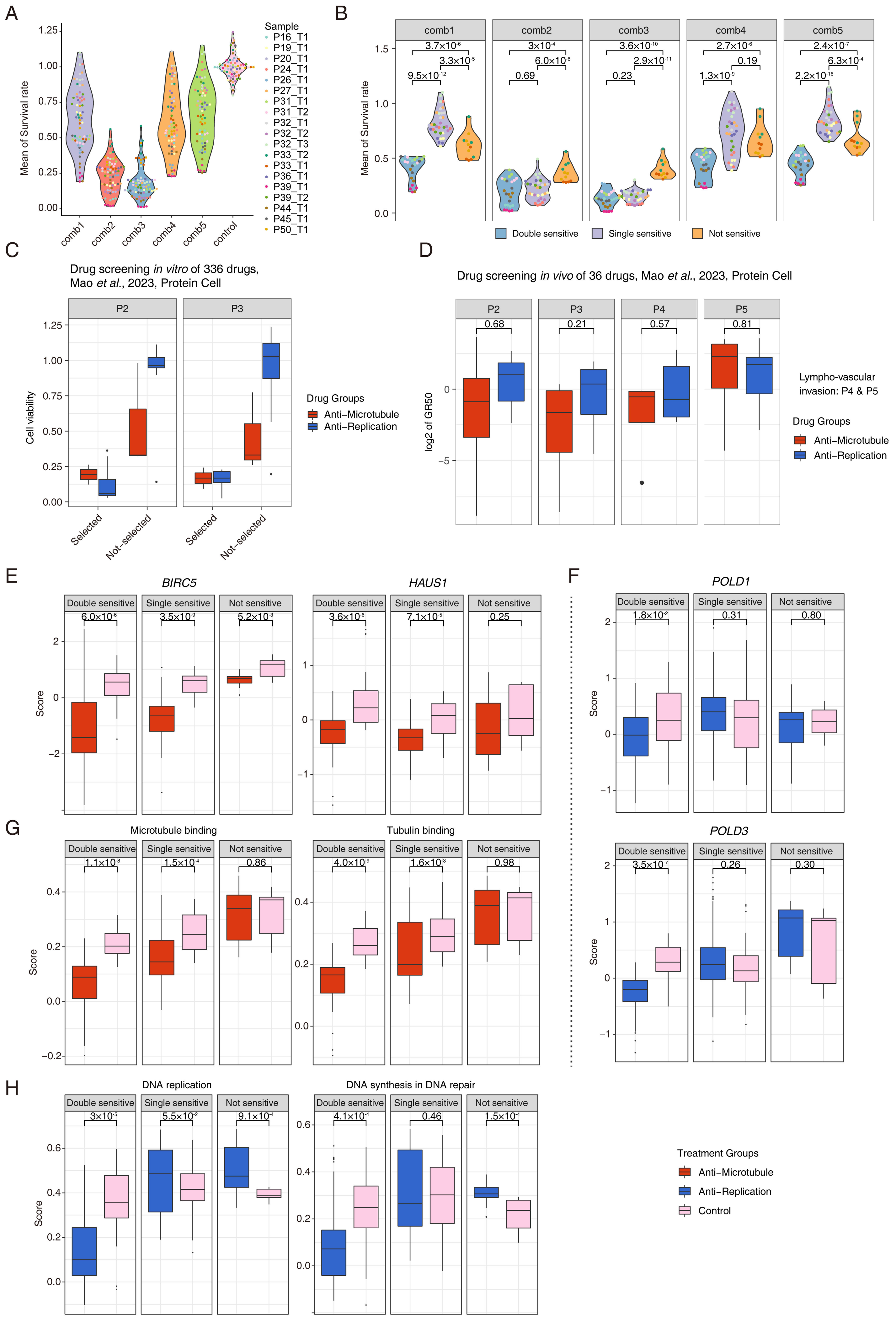
A
Mean of Survival rate
comb1
comb2
comb3
comb4
comb5
control
Sample
P16_T1
P19_T1
P20_T1
P24_T1
P26_T1
P27_T1
P31_T1
P31_T2
P32_T1
P32_T2
P32_T3
P33_T2
P33_T1
P33_T2
P36_T1
P39_T1
P39_T2
P44_T1
P45_T1
P50_T1
B
Mean of Survival rate
Double sensitive
Single sensitive
Not sensitive
C
Drug screening *in vitro* of 336 drugs, Mao *et al*., 2023, Protein Cell
P2
P3
Cell viability
Selected
Not-selected
Drug Groups
Anti−Microtubule
Anti−Replication
D
Drug screening *in vivo* of 36 drugs, Mao *et al*., 2023, Protein Cell
P2
P3
P4
P5
log2 of GR50
Lympho-vascular invasion: P4 & P5
Drug Groups
Anti−Microtubule
Anti−Replication
E
BIRC5
HAUS1
Double sensitive
Single sensitive
Not sensitive
Score
F
POLD1
POLD3
Score
G
Microtubule binding
Tubulin binding
Score
H
DNA replication
DNA synthesis in DNA repair
Score
Treatment Groups
Anti−Microtubule
Anti−Replication
Control

Figure S4. Cell viabilities and signatures in different response groups.

A. Mean cell viabilities of PDOs by different treatments.
B. Mean of cell viabilities for PDOs in each treatment.
C. Cell viabilities of selected and unselected small-molecule drugs for P2 and P3 of a previous study. P, patient.
D. Cell viabilities of the anti-microtubule and anti-replication drugs for P2, P3, P4, P5 of a previous study. P, patient.
E. The expression of *BIRC5* and *HAUS1* after the anti-microtubule treatments in the 3 response groups.
F. The expression of *POLD1* and *POLD3* after the anti-replication treatments in the 3 response groups.
G. The scores of the microtubule binding geneset and the tubulin binding geneset after the anti-microtubule treatments in the 3 response groups.
H. The scores of the DNA replication geneset and the DNA synthesis in DNA repair geneset after the anti-microtubule treatments in the 3 response groups.

Figure S5

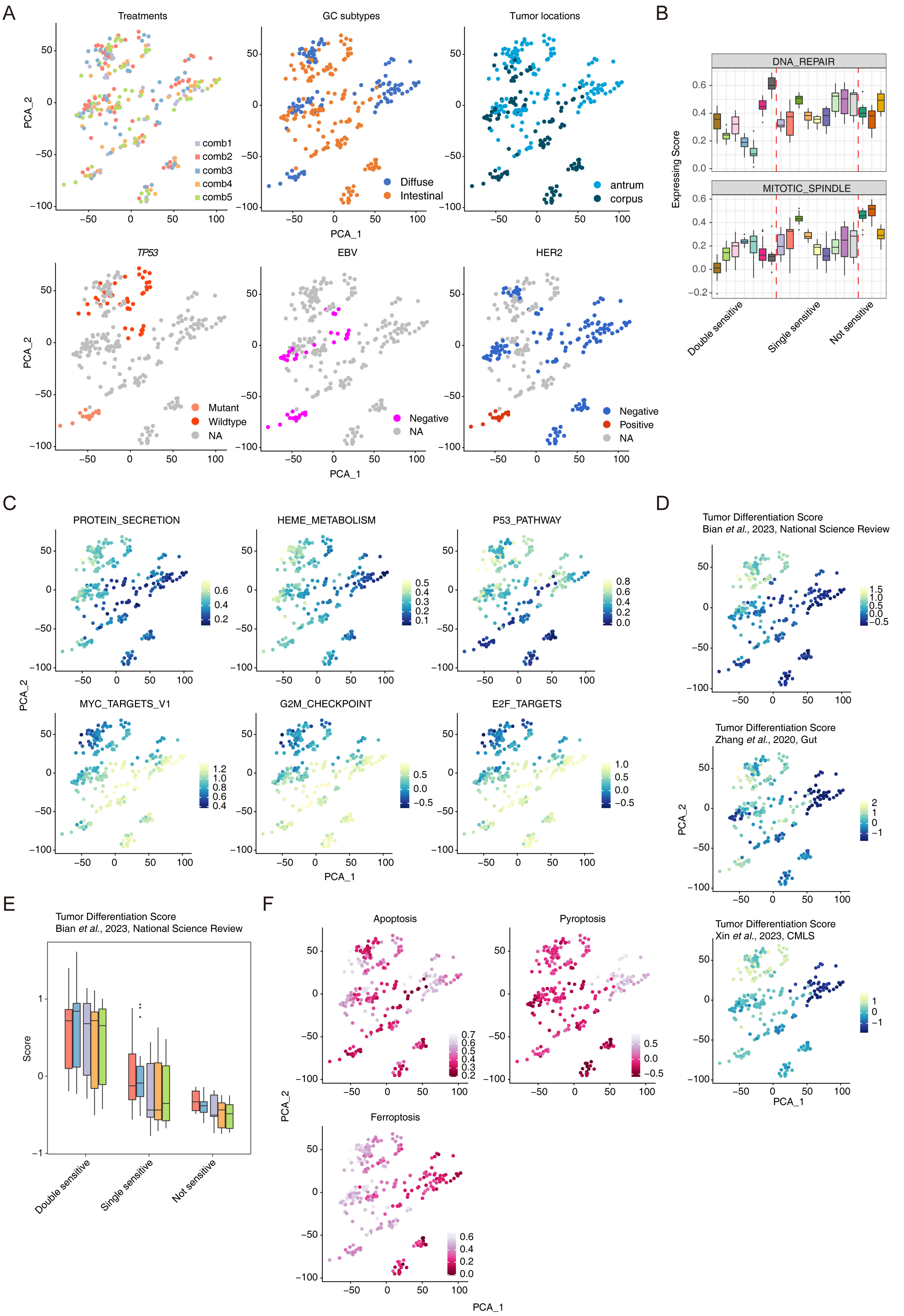
A
Treatments
GC subtypes
Tumor locations
comb1
comb2
comb3
comb4
comb5
Diffuse
Intestinal
antrum
corpus
TP53
EBV
HER2
Mutant
Wildtype
NA
Negative
NA
Negative
Positive
NA
PCA_1
PCA_2
B
DNA_REPAIR
MITOTIC_SPINDLE
Expressing Score
Double sensitive
Single sensitive
Not sensitive
C
PROTEIN_SECRETION
HEME_METABOLISM
P53_PATHWAY
MYC_TARGETS_V1
G2M_CHECKPOINT
E2F_TARGETS
D
Tumor Differentiation Score
Bian et al., 2023, National Science Review
Tumor Differentiation Score
Zhang et al., 2020, Gut
Tumor Differentiation Score
Xin et al., 2023, CMLS
E
Tumor Differentiation Score
Bian et al., 2023, National Science Review
Score
Double sensitive
Single sensitive
Not sensitive
F
Apoptosis
Pyroptosis
Ferroptosis

Figure S5. Features of treated tumor PDOs.

A. The scatterplots of PCA about all the treated PDOs with different annotations, including treatments, GC subtypes, tumor locations, *TP53* mutation state, EBV-positive state and HER2-positive state.
B. The expression scores of geneset signatures of PDOs from the 3 response groups.
C. The scatterplots of PCA about all the treated PDOs show the scores of the hallmark genesets.
D. The scatterplots of PCA about all the treated PDOs show the scores of tumor differentiation genesets.
E. The boxplot shows the scores of apoptosis, ferroptosis, and pyroptosis genesets for all the treated PDOs.
F. The scatterplots of PCA about all the treated PDOs show the scores of apoptosis, ferroptosis and pyroptosis genesets. The colors indicate the gene expression level.

Figure S6

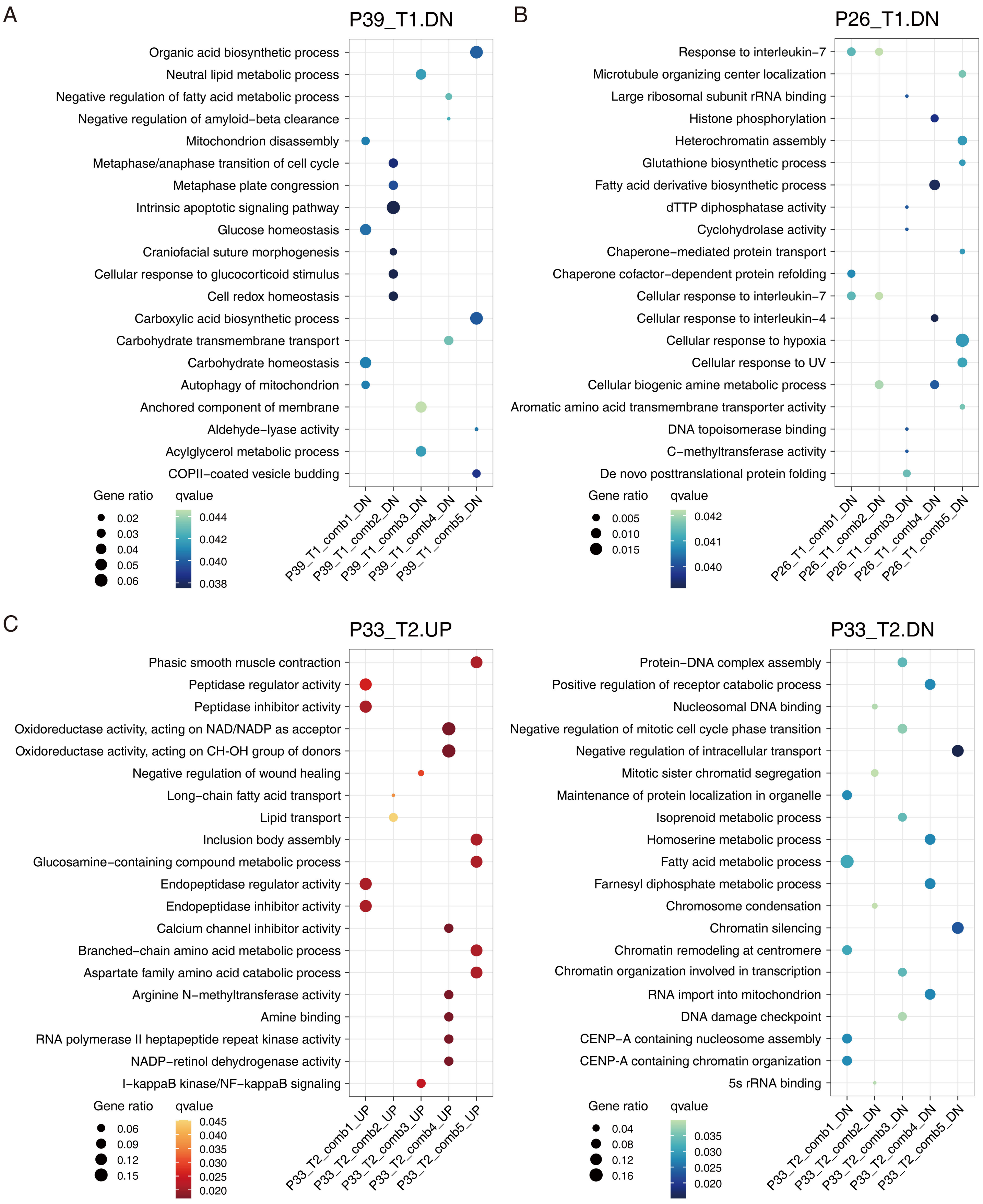

A
P39_T1.DN
Organic acid biosynthetic process
Neutral lipid metabolic process
Negative regulation of fatty acid metabolic process
Negative regulation of amyloid−beta clearance
Mitochondrion disassembly
Metaphase/anaphase transition of cell cycle
Metaphase plate congression
Intrinsic apoptotic signaling pathway
Glucose homeostasis
Craniofacial suture morphogenesis
Cellular response to glucocorticoid stimulus
Cell redox homeostasis
Carboxylic acid biosynthetic process
Carbohydrate transmembrane transport
Carbohydrate homeostasis
Autophagy of mitochondrion
Anchored component of membrane
Aldehyde−lyase activity
Acylglycerol metabolic process
COPII−coated vesicle budding
Gene ratio
0.02
0.03
0.04
0.05
0.06
qvalue
0.044
0.042
0.040
0.038
P39_T1_comb1_DN
P39_T1_comb2_DN
P39_T1_comb3_DN
P39_T1_comb4_DN
P39_T1_comb5_DN
B
P26_T1.DN
Response to interleukin−7
Microtubule organizing center localization
Large ribosomal subunit rRNA binding
Histone phosphorylation
Heterochromatin assembly
Glutathione biosynthetic process
Fatty acid derivative biosynthetic process
dTTP diphosphatase activity
Cyclohydrolase activity
Chaperone−mediated protein transport
Chaperone cofactor−dependent protein refolding
Cellular response to interleukin−7
Cellular response to interleukin−4
Cellular response to hypoxia
Cellular response to UV
Cellular biogenic amine metabolic process
Aromatic amino acid transmembrane transporter activity
DNA topoisomerase binding
C−methyltransferase activity
De novo posttranslational protein folding
Gene ratio
0.005
0.010
0.015
qvalue
0.042
0.041
0.040
P26_T1_comb1_DN
P26_T1_comb2_DN
P26_T1_comb3_DN
P26_T1_comb4_DN
P26_T1_comb5_DN
C
P33_T2.UP
Phasic smooth muscle contraction
Peptidase regulator activity
Peptidase inhibitor activity
Oxidoreductase activity, acting on NAD/NADP as acceptor
Oxidoreductase activity, acting on CH-OH group of donors
Negative regulation of wound healing
Long−chain fatty acid transport
Lipid transport
Inclusion body assembly
Glucosamine−containing compound metabolic process
Endopeptidase regulator activity
Endopeptidase inhibitor activity
Calcium channel inhibitor activity
Branched−chain amino acid metabolic process
Aspartate family amino acid catabolic process
Arginine N−methyltransferase activity
Amine binding
RNA polymerase II heptapeptide repeat kinase activity
NADP−retinol dehydrogenase activity
I−kappaB kinase/NF−kappaB signaling
Gene ratio
0.06
0.09
0.12
0.15
qvalue
0.045
0.040
0.035
0.030
0.025
0.020
P33_T2_comb1_UP
P33_T2_comb2_UP
P33_T2_comb3_UP
P33_T2_comb4_UP
P33_T2_comb5_UP
P33_T2.DN
Protein−DNA complex assembly
Positive regulation of receptor catabolic process
Nucleosomal DNA binding
Negative regulation of mitotic cell cycle phase transition
Negative regulation of intracellular transport
Mitotic sister chromatid segregation
Maintenance of protein localization in organelle
Isoprenoid metabolic process
Homoserine metabolic process
Fatty acid metabolic process
Farnesyl diphosphate metabolic process
Chromosome condensation
Chromatin silencing
Chromatin remodeling at centromere
Chromatin organization involved in transcription
RNA import into mitochondrion
DNA damage checkpoint
CENP−A containing nucleosome assembly
CENP-A containing chromatin organization
5s rRNA binding
Gene ratio
0.04
0.08
0.12
0.16
qvalue
0.035
0.030
0.025
0.020
P33_T2_comb1_DN
P33_T2_comb2_DN
P33_T2_comb3_DN
P33_T2_comb4_DN
P33_T2_comb5_DN

Figure S6. Altered signatures after treatments of PDOs.

A. Down-regulated enriched terms in P39_T1 compared with untreated PDOs.
B. Down-regulated enriched terms in P26_T1 compared with untreated PDOs.
C. Up-regulated enriched terms in P33_T2 compared with untreated PDOs.
D. Down-regulated enriched terms in P33_T2 compared with untreated PDOs

Figure S7

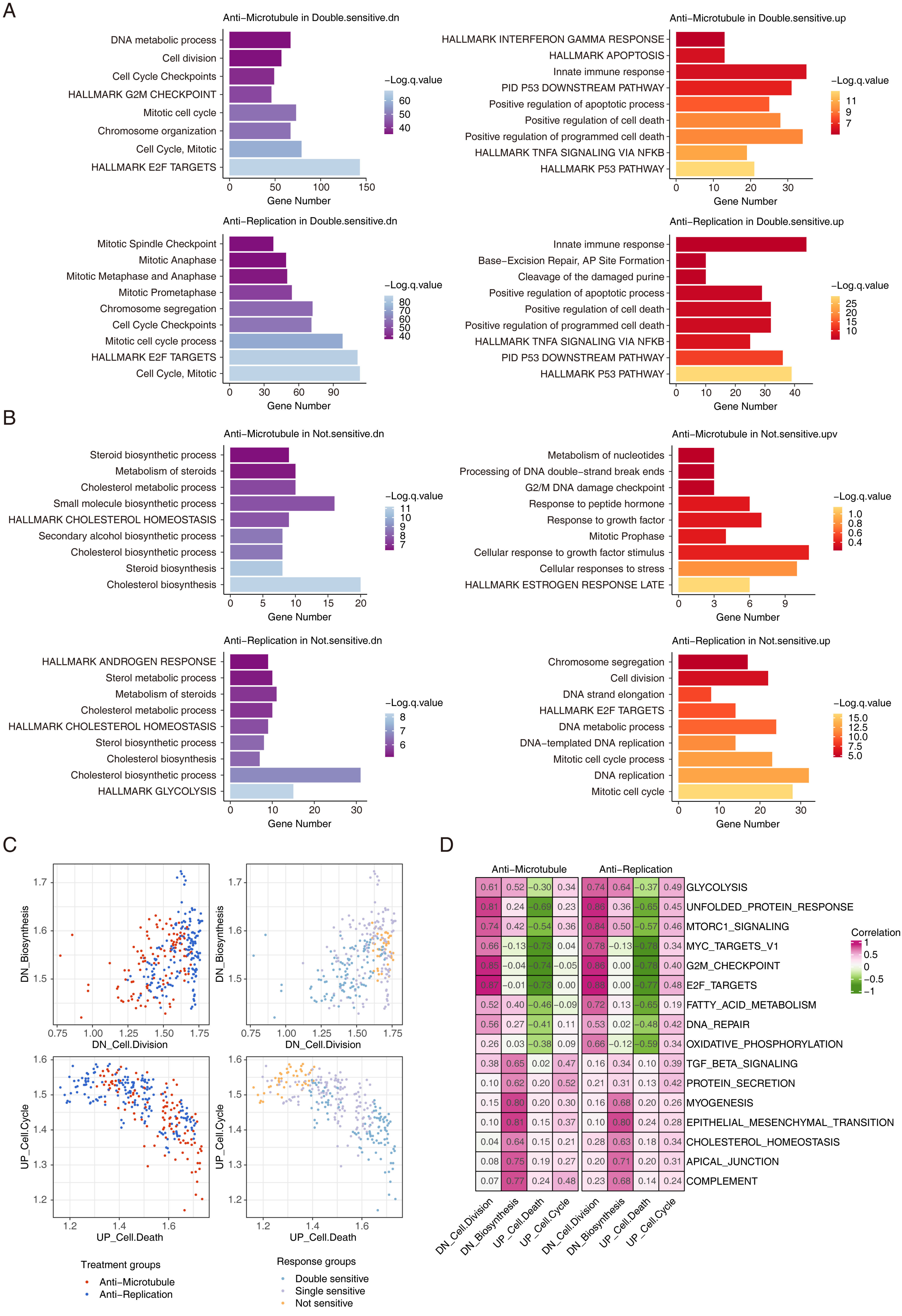
A
Anti−Microtubule in Double.sensitive.dn
DNA metabolic process
Cell division
Cell Cycle Checkpoints
HALLMARK G2M CHECKPOINT
Mitotic cell cycle
Chromosome organization
Cell Cycle, Mitotic
HALLMARK E2F TARGETS
Gene Number
−Log.q.value
Anti−Microtubule in Double.sensitive.up
HALLMARK INTERFERON GAMMA RESPONSE
HALLMARK APOPTOSIS
Innate immune response
PID P53 DOWNSTREAM PATHWAY
Positive regulation of apoptotic process
Positive regulation of cell death
Positive regulation of programmed cell death
HALLMARK TNFA SIGNALING VIA NFKB
HALLMARK P53 PATHWAY
Anti−Replication in Double.sensitive.dn
Mitotic Spindle Checkpoint
Mitotic Anaphase
Mitotic Metaphase and Anaphase
Mitotic Prometaphase
Chromosome segregation
Cell Cycle Checkpoints
Mitotic cell cycle process
HALLMARK E2F TARGETS
Cell Cycle, Mitotic
Anti−Replication in Double.sensitive.up
Innate immune response
Base−Excision Repair, AP Site Formation
Cleavage of the damaged purine
Positive regulation of apoptotic process
Positive regulation of cell death
Positive regulation of programmed cell death
HALLMARK TNFA SIGNALING VIA NFKB
PID P53 DOWNSTREAM PATHWAY
HALLMARK P53 PATHWAY
B
Anti−Microtubule in Not.sensitive.dn
Steroid biosynthetic process
Metabolism of steroids
Cholesterol metabolic process
Small molecule biosynthetic process
HALLMARK CHOLESTEROL HOMEOSTASIS
Secondary alcohol biosynthetic process
Cholesterol biosynthetic process
Steroid biosynthesis
Cholesterol biosynthesis
Anti−Microtubule in Not.sensitive.upv
Metabolism of nucleotides
Processing of DNA double−strand break ends
G2/M DNA damage checkpoint
Response to peptide hormone
Response to growth factor
Mitotic Prophase
Cellular response to growth factor stimulus
Cellular responses to stress
HALLMARK ESTROGEN RESPONSE LATE
Anti−Replication in Not.sensitive.dn
HALLMARK ANDROGEN RESPONSE
Sterol metabolic process
Metabolism of steroids
Cholesterol metabolic process
HALLMARK CHOLESTEROL HOMEOSTASIS
Sterol biosynthetic process
Cholesterol biosynthesis
Cholesterol biosynthetic process
HALLMARK GLYCOLYSIS
Anti−Replication in Not.sensitive.up
Chromosome segregation
Cell division
DNA strand elongation
HALLMARK E2F TARGETS
DNA metabolic process
DNA−templated DNA replication
Mitotic cell cycle process
DNA replication
Mitotic cell cycle
C
DN_Biosynthesis
DN_Cell.Division
UP_Cell.Cycle
UP_Cell.Death
Treatment groups
Anti−Microtubule
Anti−Replication
Response groups
Double sensitive
Single sensitive
Not sensitive
D
Anti−Microtubule
Anti−Replication
0.61 0.52 −0.30 0.34 0.74 0.64 −0.37 0.49 GLYCOLYSIS
0.81 0.24 −0.69 0.23 0.86 0.36 −0.65 0.45 UNFOLDED_PROTEIN_RESPONSE
0.74 0.42 −0.54 0.36 0.84 0.50 −0.57 0.46 MTORC1_SIGNALING
0.66 −0.13 −0.73 0.04 0.78 −0.13 −0.78 0.34 MYC_TARGETS_V1
0.85 −0.04 −0.74 −0.05 0.86 0.00 −0.78 0.40 G2M_CHECKPOINT
0.87 −0.01 −0.73 0.00 0.86 0.00 −0.77 0.48 E2F_TARGETS
0.52 0.40 −0.46 −0.09 0.72 0.13 −0.65 0.19 FATTY_ACID_METABOLISM
0.56 0.27 −0.41 0.11 0.53 0.02 −0.48 0.42 DNA_REPAIR
0.26 0.03 −0.38 0.09 0.66 −0.12 −0.59 0.34 OXIDATIVE_PHOSPHORYLATION
0.38 0.65 0.02 0.47 0.16 0.34 0.10 0.39 TGF_BETA_SIGNALING
0.10 0.62 0.20 0.52 0.21 0.31 0.13 0.42 PROTEIN_SECRETION
0.15 0.80 0.20 0.30 0.16 0.68 0.20 0.26 MYOGENESIS
0.10 0.81 0.15 0.37 0.10 0.80 0.24 0.28 EPITHELIAL_MESENCHYMAL_TRANSITION
0.04 0.64 0.15 0.21 0.28 0.63 0.18 0.34 CHOLESTEROL_HOMEOSTASIS
0.08 0.75 0.19 0.27 0.20 0.71 0.20 0.31 APICAL_JUNCTION
0.07 0.77 0.24 0.48 0.23 0.68 0.14 0.24 COMPLEMENT
DN_Cell.Division
DN_Biosynthesis
UP_Cell.Death
UP_Cell.Cycle
Correlation

Figure S7. The 4 commonly altered signatures after the anti-microtubule treatments and the anti-replication treatments.

A. The down-regulated enriched terms of the anti-microtubule treatments and the anti-replication treatments in the double-sensitive group.
B. The down-regulated enriched terms of the anti-microtubule treatments and the anti-replication treatments in the not-sensitive group.
C. The scatterplots show the scores of these above altered gene signatures.
D. The correlation of the hallmark genesets and these above enriched signatures in the anti-microtubule treatments and the anti-replication treatments.

Figure S8

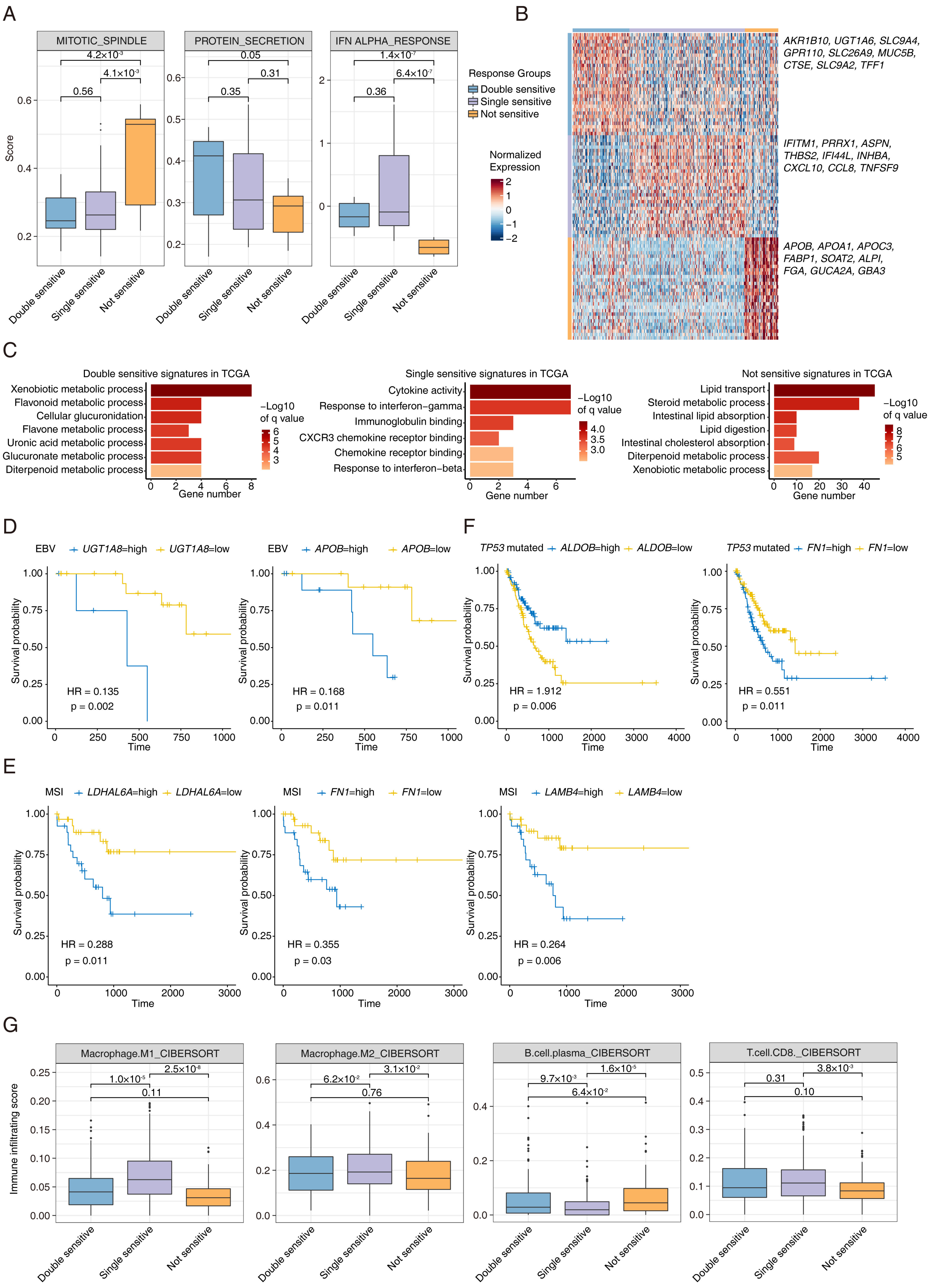

A
MITOTIC_SPINDLE
PROTEIN_SECRETION
IFN ALPHA_RESPONSE
Score
Double sensitive
Single sensitive
Not sensitive
Response Groups
Double sensitive
Single sensitive
Not sensitive
B
Normalized Expression
AKR1B10, UGT1A6, SLC9A4, GPR110, SLC26A9, MUC5B, CTSE, SLC9A2, TFF1
IFITM1, PRRX1, ASPN, THBS2, IFI44L, INHBA, CXCL10, CCL8, TNFSF9
APOB, APOA1, APOC3, FABP1, SOAT2, ALPI, FGA, GUCA2A, GBA3
C
Double sensitive signatures in TCGA
Xenobiotic metabolic process
Flavonoid metabolic process
Cellular glucuronidation
Flavone metabolic process
Uronic acid metabolic process
Glucuronate metabolic process
Diterpenoid metabolic process
Single sensitive signatures in TCGA
Cytokine activity
Response to interferon−gamma
Immunoglobulin binding
CXCR3 chemokine receptor binding
Chemokine receptor binding
Response to interferon−beta
Not sensitive signatures in TCGA
Lipid transport
Steroid metabolic process
Intestinal lipid absorption
Lipid digestion
Intestinal cholesterol absorption
Diterpenoid metabolic process
Xenobiotic metabolic process
−Log10 of q value
Gene number
D
EBV UGT1A8=high UGT1A8=low
HR = 0.135
p = 0.002
EBV APOB=high APOB=low
HR = 0.168
p = 0.011
F
TP53 mutated ALDOB=high ALDOB=low
HR = 1.912
p = 0.006
TP53 mutated FN1=high FN1=low
HR = 0.551
p = 0.011
Survival probability
Time
E
MSI LDHAL6A=high LDHAL6A=low
HR = 0.288
p = 0.011
MSI FN1=high FN1=low
HR = 0.355
p = 0.03
MSI LAMB4=high LAMB4=low
HR = 0.264
p = 0.006
G
Macrophage.M1_CIBERSORT
Macrophage.M2_CIBERSORT
B.cell.plasma_CIBERSORT
T.cell.CD8._CIBERSORT
Immune infiltrating score

Figure S8. The expression and molecular subtypes related signatures of the predicted response groups in the TCGA dataset.

A. The expression of hallmark genesets in the 3 response groups of all the untreated samples.
B. DEGs of identified response groups in GC samples from the TCGA dataset.
C. Enriched terms of identified response groups in GC samples from the TCGA dataset.
D. Prognostic markers in the EBV-positive subtype. Survival curves showing the overall survival probability of EBV-positive patients stratified by the expression levels (high vs. low) of *UGT1A8* and *APOB*.
E. Prognostic markers in the MSI-high subtype. Survival curves showing the overall survival probability of MSI-high patients stratified by the expression levels (high vs. low) of *LDHAL6A*, *FN1*, and *LAMB4*.
F. Prognostic markers in the *TP53*-mutated subtype. Survival curves showing the overall survival probability of *TP53*-mutated patients stratified by the expression levels (high vs. low) of *ALDOB* and *FN1*.
G. Inferred cell proportions of immune cell types in the identified response groups of GC samples from the TCGA dataset.

Figure S9

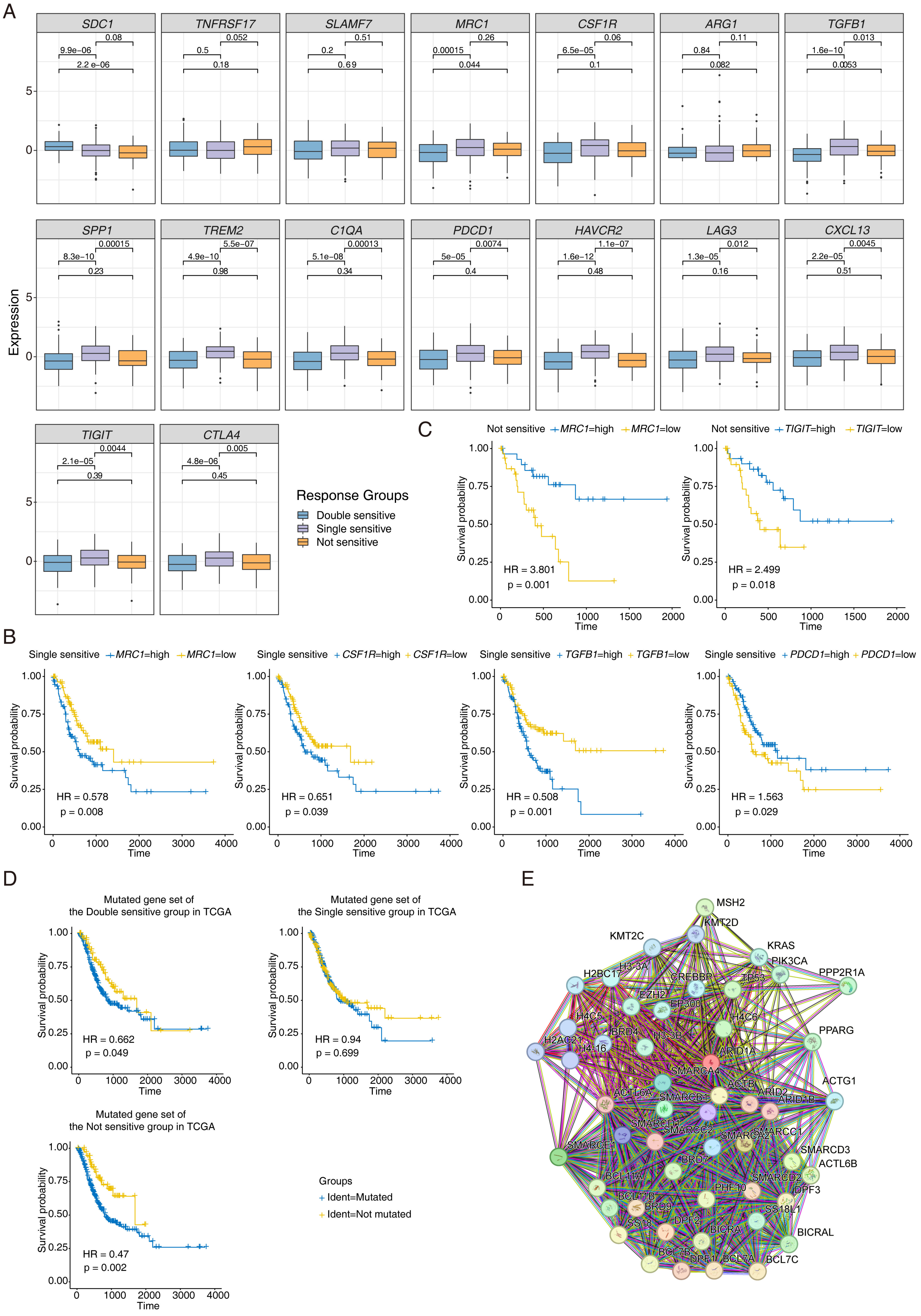
A
SDC1
TNFRSF17
SLAMF7
MRC1
CSF1R
ARG1
TGFB1
SPP1
TREM2
C1QA
PDCD1
HAVCR2
LAG3
CXCL13
TIGIT
CTLA4
Expression
Response Groups
Double sensitive
Single sensitive
Not sensitive
B
Single sensitive MRC1=high MRC1=low
HR = 0.578
p = 0.008
Single sensitive CSF1R=high CSF1R=low
HR = 0.651
p = 0.039
Single sensitive TGFB1=high TGFB1=low
HR = 0.508
p = 0.001
Single sensitive PDCD1=high PDCD1=low
HR = 1.563
p = 0.029
Survival probability
Time
C
Not sensitive MRC1=high MRC1=low
HR = 3.801
p = 0.001
Not sensitive TIGIT=high TIGIT=low
HR = 2.499
p = 0.018
D
Mutated gene set of the Double sensitive group in TCGA
HR = 0.662
p = 0.049
Mutated gene set of the Single sensitive group in TCGA
HR = 0.94
p = 0.699
Mutated gene set of the Not sensitive group in TCGA
HR = 0.47
p = 0.002
Groups
Ident=Mutated
Ident=Not mutated
E
MSH2
KMT2D
KMT2C
KRAS
PIK3CA
PPP2R1A
H2BC17
H3-3A
CREBBP
TP53
EZH2
EP300
H4C5
H4C6
BRD4
H3-3B
H2AC21
H4-16
ARID1A
PPARG
SMARCA4
ACTB
ACTG1
ACTL6A
SMARCB1
ARID2
ARID1B
SMARCD1
SMARCC2
SMARCA2
SMARCC1
SMARCE1
SMARCD3
BRD7
ACTL6B
BCL11A
SMARCD2
PHF10
DPF3
BCL11B
BRD9
SS18L1
SS18
DPF2
BICRA
BICRAL
BCL7B
DPF1
BCL7A
BCL7C

Figure S9. The immune and mutation signatures of the predicted response groups in the TCGA dataset.

A. Expression of functional immune markers. Boxplots showing the expression levels of specific marker genes for plasma cells, macrophages, and exhausted $CD8^+$ T cells across the three response groups.
B. Prognostic value of immune markers in the single-sensitive group. Survival curves showing the association between the expression of *MRC1*, *CSF1R*, *TGFB1*, and *PDCD1* and patient overall survival within the single-sensitive group.
C. Prognostic value of immune markers in the not-sensitive group. Survival curves showing the association between the expression of *MRC1* and *TIGIT* and patient overall survival within the not-sensitive group.
D. The survival analysis of identified enriched mutational genesets in the identified response groups of GC samples from the TCGA dataset.
E. The protein-protein interaction network of ARID1A with other proteins from the StringDB database.

Figure S10

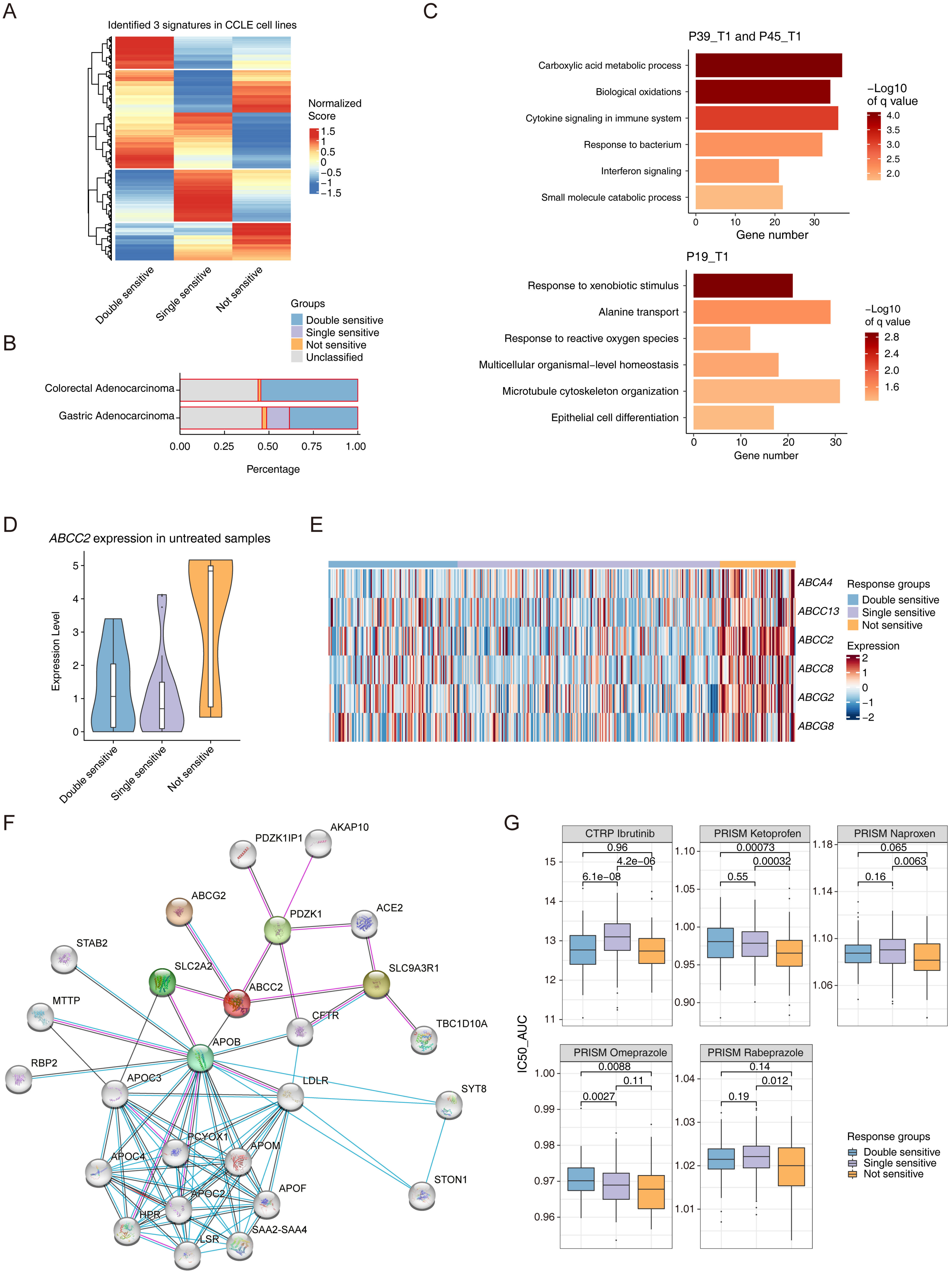
A
Identified 3 signatures in CCLE cell lines
Normalized Score
1.5
1
0.5
0
−0.5
−1
−1.5
Double sensitive
Single sensitive
Not sensitive
B
Groups
Double sensitive
Single sensitive
Not sensitive
Unclassified
Colorectal Adenocarcinoma
Gastric Adenocarcinoma
0.00
0.25
0.50
0.75
1.00
Percentage
C
P39_T1 and P45_T1
Carboxylic acid metabolic process
Biological oxidations
Cytokine signaling in immune system
Response to bacterium
Interferon signaling
Small molecule catabolic process
Gene number
−Log10 of q value
P19_T1
Response to xenobiotic stimulus
Alanine transport
Response to reactive oxygen species
Multicellular organismal−level homeostasis
Microtubule cytoskeleton organization
Epithelial cell differentiation
Gene number
−Log10 of q value
D
ABCC2 expression in untreated samples
Expression Level
Double sensitive
Single sensitive
Not sensitive
E
ABCA4
ABCC13
ABCC2
ABCC8
ABCG2
ABCG8
Response groups
Double sensitive
Single sensitive
Not sensitive
Expression
F
PDZK1IP1
AKAP10
ABCG2
PDZK1
ACE2
STAB2
SLC2A2
ABCC2
SLC9A3R1
MTTP
CFTR
TBC1D10A
APOB
RBP2
APOC3
LDLR
SYT8
PCYOX1
APOM
APOC4
APOC2
APOF
STON1
HPR
SAA2-SAA4
LSR
G
CTRP Ibrutinib
0.96
4.2e−06
6.1e−08
PRISM Ketoprofen
0.00073
0.00032
0.55
PRISM Naproxen
0.065
0.0063
0.16
IC50_AUC
PRISM Omeprazole
0.0088
0.11
0.0027
PRISM Rabeprazole
0.14
0.012
0.19
Response groups
Double sensitive
Single sensitive
Not sensitive

Figure S10. The classification of the above 3 response groups from the CCLE dataset and ABCC2 associated analysis.

A. The classification of cell lines from the CCLE dataset based on these 3 signatures.
B. The proportion of these 3 response groups in different types of cancer cell lines.
C. The bar plot shows the enrichment of different signatures in P39_T1 and P45_T1 or P19_T1.
D. Expression of *ABCC2* in all the untreated PDOs of 3 response groups.
E. Expression of ABC family genes in classified groups from TCGA dataset.
F. The protein-protein interaction network of ABCC2 from the StringDB database.
G. Predicted drug sensitivity of MRP-modulating agents. Boxplots showing the predicted IC50 values (AUC) of ibrutinib, ketoprofen, naproxen, omeprazole, and rabeprazole across the three response groups.

Supplementary tables

Table S1: Clinical information of GC patients in this study.

Table S2: Cell viability of 5 combination treatments for PDOs from different patients.

Table S3: Genesets used for scoring the treated PDOs in this study.

Table S4: Differentially expressed enriched signatures of different combination treatments.
